# Differential Production of Psl in Planktonic Cells Leads to Two Distinctive Attachment Phenotypes in Pseudomonas aeruginosa

**DOI:** 10.1128/AEM.00700-18

**Published:** 2018-07-02

**Authors:** Shuai Yang, Xinyi Cheng, Zhenyu Jin, Aiguo Xia, Lei Ni, Rongrong Zhang, Fan Jin

**Affiliations:** a Hefei National Laboratory for Physical Sciences at the Microscale, University of Science and Technology of China, Hefei, People's Republic of China; b Department of Polymer Science and Engineering, University of Science and Technology of China, Hefei, People's Republic of China; c CAS Key Laboratory of Soft Matter Chemistry, University of Science and Technology of China, Hefei, People's Republic of China; University of Tartu

**Keywords:** Pseudomonas aeruginosa, attachment, biofilms, polysaccharides

## Abstract

Exponentially growing bacteria in a well-mixed planktonic culture are generally assumed to be physiologically and phenotypically uniform and distinct from their genetically identical counterparts living in biofilms. Using a combination of high spatiotemporal microscopy and a bacterial tracking algorithm, in this study, we showed that planktonic cells of Pseudomonas aeruginosa differently attached to surfaces even when they remained in the exponential phase. We consistently observed that fast- and slow-attaching phenotypes coexist in planktonic cells, regardless of their growth phase. Furthermore, we found that (i) the distinct attaching phenotypes of planktonic cells resulted from the differential production of Psl and (ii) the RsmYZ/RsmA signaling pathway mainly regulated the differential production of Psl. Our results indicate that the differential production of Psl in P. aeruginosa plays a significant role in biofilm development and formation.

**IMPORTANCE** The attachment of planktonic cells to surfaces is the first and most crucial step in biofilm formation. In this paper, we show that planktonic cells of Pseudomonas aeruginosa differently attach to surfaces. Typically, in the later exponential phase, approximately 80% of the cells can quickly attach to surfaces within 15 min, whereas approximately 20% of the cells slowly attach to surfaces, which greatly affects the initial stage of biofilm formation in the presence of flows. This is because fast-attaching cells are more likely to attach on surfaces to form microcolonies, whereas slow-attaching cells are more likely to remain in the mobile phase. This scenario is different from the previous understanding of biofilm formation in the initial stage, in which planktonic cells were thought to uniformly attach to surfaces. Most notably, the results of this study show that the different attachment manner of planktonic cells to surfaces affects the subsequent stages of biofilm formation. This research highlights that the phenotypic variations in planktonic cells plays significant roles in various stages of biofilm formation.

## Abstract (precis)

The attachment of planktonic cells to surfaces is the first and most crucial step in biofilm formation. In this paper, we show that planktonic cells of Pseudomonas aeruginosa differently attach to surfaces. Typically, in the later exponential phase, approximately 80% of the cells can quickly attach to surfaces within 15 min, whereas approximately 20% of the cells slowly attach to surfaces, which greatly affects the initial stage of biofilm formation in the presence of flows. This is because fast-attaching cells are more likely to attach on surfaces to form microcolonies, whereas slow-attaching cells are more likely to remain in the mobile phase. This scenario is different from the previous understanding of biofilm formation in the initial stage, in which planktonic cells were thought to uniformly attach to surfaces. Most notably, the results of this study show that the different attachment manner of planktonic cells to surfaces affects the subsequent stages of biofilm formation. This research highlights that the phenotypic variations in planktonic cells plays significant roles in various stages of biofilm formation.

## INTRODUCTION

Biofilms are typically composed of a dense layer of bacteria growing in a self-produced matrix (1) that holds the cells together to form surface-associated communities (2), thereby enabling them to survive or thrive in various environments ranging from mineral surfaces to human tissue (3, 4). Individual cells growing in biofilms are physiologically and phenotypically distinct even if the biofilms is formed by a genetically identical species (5). This is partially because of the nonhomogeneous distribution of nutrients (6), electron acceptors (oxygen) (7), metabolic waste, and signaling molecules (8) in biofilms, where the local concentrations of these small molecules are determined by their diffusion rates, cell densities, and external cycling conditions (9). In contrast, exponentially growing cells in a well-mixed planktonic culture are traditionally assumed to be physiologically and phenotypically uniform (10). Increasingly, researchers have reported cell-to-cell heterogeneity in a variety of physiological parameters, including growth rate, chemotaxis, metabolism, nutritional acquisition, and tolerance to noxious stimuli, including antibiotics (11–16), and this phenotypic heterogeneity in an isogenic population has been observed in many different bacterial species (17–25). Heterogeneity in gene expression, both from individual genes and as output from more complicated gene circuits, is mainly responsible for the phenotypic variation, and it has been shown that this variation arises from fluctuations in transcription and mRNA or protein stability and/or translation, which is a fundamental property of living systems, including eukaryotes, called stochasticity (26–28). In some cases, phenotypic heterogeneity can be attributed to population differences in the concentration of molecules such as the second messenger, c-di-GMP (18, 29); notably, the differences are not simply stochastic but involve a specific molecular mechanism (20).

Heterogeneity in gene expression, growth rate, division time, or nucleotide-based second messengers has been measured in individual cells. Coincidently, in this context, attaching phenotypic variation occurs in the planktonic cells of Pseudomonas aeruginosa, which causes them to form distinctive phenotypes in the development of biofilms, for which biofilm formation generally starts with bacterial attachment to a surface (30).

Ni et al. showed that P. aeruginosa cells differently deploy their type IV pili in a unique manner to mediate distinctive twitching motilities with a mobile or immobile phenotype after the initial attachment of planktonic cells (31). Based on these findings, we aimed to determine whether such planktonic cells are indeed phenotypically uniform, as commonly believed. Thus, in this study, we investigated how the planktonic cells of P. aeruginosa attach to surfaces by using a combination of high spatiotemporal microscopy and a bacterial tracking algorithm (32, 33). We consistently observed that the planktonic cells differently attached to surfaces, regardless of their growth phase; typically, in the later exponential phase, approximately 80% of planktonic cells could quickly attach to the surface, whereas approximately 20% of cells slowly attached to the surface. Subsequently, we investigated the main molecular mechanism responsible for this phenotypic variation in planktonic cells. Our results elucidated that the RsmYZ/RsmA signaling pathway (34) differentially regulated the production of an exopolysaccharide Psl (35) in planktonic cells of P. aeruginosa, thereby enabling them to differently attach on the surface. Furthermore, we examined whether this distinctive phenotypical trait of planktonic cells affects subsequent biofilm formation. Our results indicated that the differential production of Psl in P. aeruginosa plays a significant role in various stages of biofilm formation.

## RESULTS AND DISCUSSION

### Planktonic cells of P. aeruginosa differently attach to surfaces.

We collected the planktonic cells of P. aeruginosa from a well-mixed culture at different culturing times, and their optical density at 600 nm (OD_600_) was measured. The growth curve can be used to identify the growth phase of cells (see Fig. S1 in the supplemental material). Therefore, the growth phase can be determined based on the OD_600_, as follows: early exponential phase, OD_600_ ≤ 0.6; exponential phase, 0.6 < OD_600_ ≤ 0.8; later exponential phase, 0.8 < OD_600_ ≤ 1.2; early stationary phase, 1.2 < OD_600_ ≤ 1.8; and later stationary phase, 1.8 < OD_600_ ≤ 2.2. We recognized that the stationary phase starts from the decrease of growth rate based on the definition by Kolter et al. (36). The cells were diluted to an OD_600_ of approximately 0.02 for attachment experiments (additional details are provided in Materials and Methods). By counting the surface-attached cells [*N_s_*(*t*)] over time (*t*) for a total time of about 1 h, we examined how these planktonic cells collected in various growth phases attach to a glass surface. Notably, for the attachment experiments, we did not add any carbon source to the medium to ensure that the cells could not divide in the bulk or on the surface. To further confirm that, the diluted cells were sandwiched between an FAB agarose slab without a carbon source and an imaging dish glass to monitor the cells' behavior (see “Agarose slab experiments” in Materials and Methods). The results show that the cells hardly grew in medium without carbon source in 1 h and the numbers remained constant, indicated by the fact that no division events were detected in this period (Fig. S2). Additionally, using propidium iodide (PI) staining, we found that the number of dead cells was negligible and no increase of the death rate was observed when cells were deprived of the carbon source for 1 h (Fig. S3). Therefore, this experimental condition enabled the total number of cells [*N* = *N_s_*(*t*) + *N_b_*(*t*)] to remain constant, where *N_s_*(*t*) is the number of cells attached on the surface and *N_b_*(*t*) represents the planktonic cells in the bulk at a certain time. Based on the simplification that the number of detaching cells is negligible (Fig. S4), we investigated the cells' attachment behaviors by determining the attaching kinetics (see “Mathematical model” in Materials and Methods).

**FIG 1:**
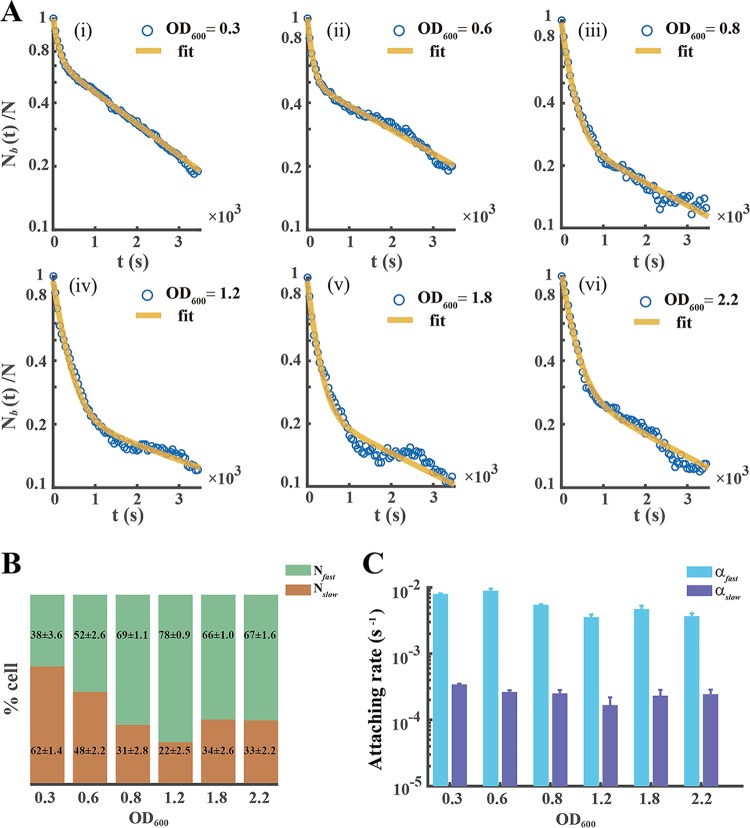
Two distinct attaching phenotypes coexist in the planktonic cells of P. aeruginosa. (A) The attachment kinetics of planktonic P. aeruginosa cells [*N_b_*(*t*)/*N*] in different growth phases always shows a two-exponent decrease. (i) lag phase (OD_600_ ∼ 0.3), (ii) early exponential phase (OD_600_ ∼ 0.6), (iii) exponential phase (OD_600_ ∼ 0.8), (iv) later exponential phase (OD_600_ ∼ 1.2), (v) stationary phase (OD_600_ ∼ 1.8), and (vi) later stationary phase (OD_600_ ∼ 2.2). The solid yellow line is a two-exponential fit to the experimental data (blue circles). (B and C) Fraction of cell numbers of the fast-attaching (*N*_fast_) or slow-attaching (*N*_slow_) phenotype (B) and corresponding attaching rate (α_fast_ or α_slow_) (C) were attained by data fitted and analyzed (supplemental material), indicating attaching character of two obviously different phenotypes. Error bars depict the standard deviations from 3 biological replicates. Experiments in panel A were carried out at least three times, and results from one representative example are shown.

Our results indicate the following. (i) The attaching kinetics of the planktonic cells of P. aeruginosa [*N_b_*(*t*)/*N*] always follow a double exponential decay, regardless of their growth phase (Fig. 1A). This shows that a fast- and slow-attaching phenotypes instinctively coexist in the planktonic cells of P. aeruginosa. (ii) The cell faction $N_{b}^{\text{fast}}/N$ that results from the fast-attaching phenotype is positively related to OD_600_ (Fig. 1B), indicating that the stationary phase contains more planktonic cells with the fast-attaching phenotype. (iii) The attaching rates contrast for the two phenotypes. In the later exponential phase, the attaching rate [α_fast_ = 5.45 × 10^−3^ s^−1^] that results from the fast-attaching phenotype is typically 20 times higher than that [α_slow_ = 2.52 × 10^−4^ s^−1^] which results from the slow-attaching phenotype (Fig. 1C). (iv) α_fast_ or α_slow_ weakly depends on the growth phase (Fig. 1C).

### Differential production of polysaccharide Psl enables planktonic P. aeruginosa to differently attach to surfaces.

**FIG 2:**
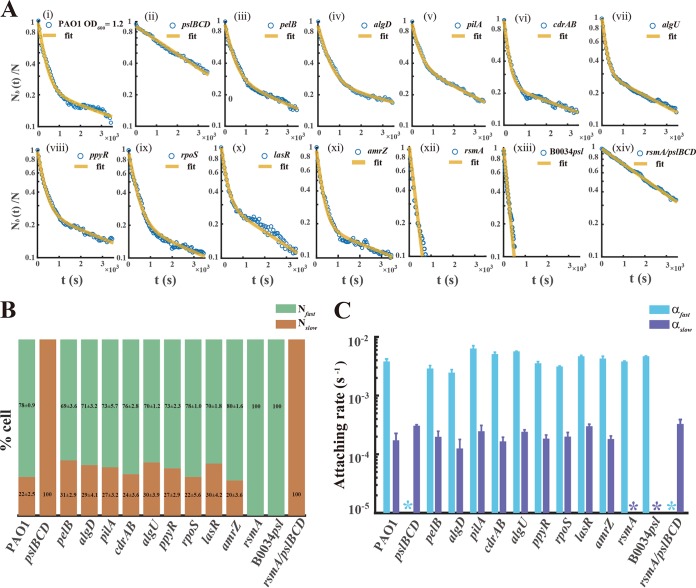
Polysaccharide Psl enables planktonic cells to differently attach to surfaces, and this is attributed to the RsmYZ/RsmA regulational pathway. (A) Attachment kinetics of planktonic cells [*N_b_*(*t*)/*N*] of P. aeruginosa mutant strains. Each mutant has no discernible effect on cell growth (Fig. S5), and we conducted attachment experiments for these mutants at the identical growth phase (later exponential phase, OD_600_ **∼** 1.2) as follows: for the wild type as a control (i); for adhesion factors, including different polysaccharides (Psl, Pel, or alginate), surface appendages (type IV pili), and adhesion proteins (CdrAB), which reveal that the differential production of polysaccharide Psl enables planktonic P. aeruginosa to differently attach on surfaces (ii to vi); for regulators of Psl expression, containing transcriptional regulators (*algU*, *rpoS*, *ppyR*, *lasR*, and *amrZ*) and the posttranscriptional regulator (*rsmA*), showing that a distinctive RsmA regulational pathway predominates the differential expression of Psl (vii to xii); for a nucleotide substitution strain in which the site of RsmA binding to the 5′ untranslated region (UTR) of the *psl* operon was replaced with artificial designed sequence BBa_B0034, further confirming the regulation (xiii); and for the Δ*rsmA* Δ*pslBCD* double mutant strain (xiv). (B) From left to right in the chart, the single stacked bar shows the fraction of two distinct attaching phenotypes (*N*_fast_ and *N*_slow_) in the mutant strains as described for panel A. (C) The graph contains 14 groups of two bars; each bar of one group was used for displaying the attachment rate (α_fast_ or α_slow_) of each mutant strain. The asterisks indicate where the color-marked attaching phenotype was not detected in the strain. Error bars depict the SDs from 3 biological replicates. Experiments in panel A were carried out at least three times, and results from one representative example are shown.

The surface attachment of the planktonic cells of P. aeruginosa depends on various adhesion factors, mainly surface appendages (type IV pili), different polysaccharides (Psl, Pel, or alginate) (37), and adhesion proteins (CdrA) (38). To reveal which adhesion factor enables planktonic cells to differently attach to surfaces, we screened the factors by using specific mutant strains with deficiency in production of the corresponding adhesion factor (Table 1). Figure 2Aii to vi show that the fast-attaching phenotype entirely disappeared in the planktonic cells which were deficient in production of Psl (Fig. 2Aii), whereas the fast- and slow-attaching phenotypes remained in the other mutant strains (Fig. 2Aiii to vi). Notably, the changes of $N_{b}^{\text{fast}}/N$ (Fig. 2B), α_fast_, and α_slow_ (Fig. 2C) were minor in the *pilA*, *pelB*, *algD*, and *cdrAB* mutant strains. These results indicate that Psl is an essential adhesion factor that enables planktonic cells to quickly attach to surfaces.

**TABLE 1:** Strains used in this study

Strain	Genotype, description, or relevant characteristics	Source
Escherichia coli		
Top10	F^−^ *mcrA* Δ(*mrr-hsdRMS*-*mcrBC*) φ80*lacZ*ΔM15 Δ*lacX74 recA1 araD139* Δ(*ara-leu*)*7697 galU galK rpsL*(Nal^r^) *endA1 nupG*	Invitrogen
Pseudomonas aeruginosa		
PAO1	Wild-type P. aeruginosa strain	J. D. Shrout
PAOYs1	*pslBCD* inactivated in PAO1; nonresistant	Present study
PAOYs2	*pelB* inactivated in PAO1; nonresistant	J. D. Shrout
PAOYs3	*algD* inactivated in PAO1; nonresistant	J. D. Shrout
PAOYs4	*pilA* inactivated in PAO1; nonresistant	Present study
PAOYs5	*cdrAB* inactivated in PAO1; nonresistant	Present study
PAOYs6	*algU* inactivated in PAO1; nonresistant	Present study
PAOYs7	*rpoS* inactivated in PAO1; nonresistant	Present study
PAOYs8	*ppyR* inactivated in PAO1; nonresistant	Present study
PAOYs9	*lasR* inactivated in PAO1; nonresistant	J. D. Shrout
PAOYs10	*amrZ* inactivated in PAO1; nonresistant	Present study
PAOYs11	*rsmA* inactivated in PAO1; nonresistant	Present study
PAOYs12	*rsmA* and *pslBCD* inactivated in PAO1; nonresistant	Present study
B0034*psl*-PAO1	PAO1 in which the RBS region of *psl* operon was replaced with a constitutive prokaryotic RBS BBa_B0034 (iGEM biobrick); nonresistant	Present study
PAO1::RYS-1	PAO1 containing a gene encoding EGFP directly under the control of the *psl* promoter integrated at the *att*Tn*7* site; Gm^r^	Present study
PAO1::RYS-2	Strain for amplifying signal. PAO1 integrated by the inverted Psl expression reporter RYS-2 at the *att*Tn*7* site; Gm^r^	Present study
*pslBCD*::RYS-2	Δ*pslBCD* integrated by RYS-2 at the *att*Tn*7* site; Gm^r^	Present study
B0034*psl*::RYS-3	Δ*pslBCD* integrated by RYS-3 at the *att*Tn*7* site; Gm^r^	Present study
PAO1::RYS-1a	Gene marker of Gm^r^ of PAO1::RYS-1 is excised; nonresistant	Present study
PAO1::RYS-2a	Gene marker of Gm^r^ of PAO1::RYS-2 is excised; nonresistant	Present study
*pslBCD*::RYS-2a	Gene marker of Gm^r^ of *pslBCD*::RYS-2 is excised; nonresistant	Present study
B0034*psl*::RYS-3a	Gene marker of Gm^r^ of B0034*psl*::RYS-3 is excised; nonresistant	Present study
PAO1::RYS-4	PAO1 harboring the RsmY reporter, RYS-4; Gm^r^	Present study
PAO1::RYS-5	PAO1 harboring the RsmZ reporter, RYS-5; Gm^r^	Present study
PAO1::RYS-6	PAO1 harboring the RsmA reporter, RYS-6; Gm^r^	Present study
PAO1-*egfp*	PAO1 tagged by EGFP integrated at the *att*Tn*7* site; Gm^r^	Present study
B0034*psl-egfp*	B0034psl-PAO1 tagged by EGFP at the *att*Tn*7* site; Gm^r^	Present study
*pslBCD-egfp*	Δ*pslBCD* tagged by EGFP at the *att*Tn*7* site; Gm^r^	Present study

**FIG 3:**
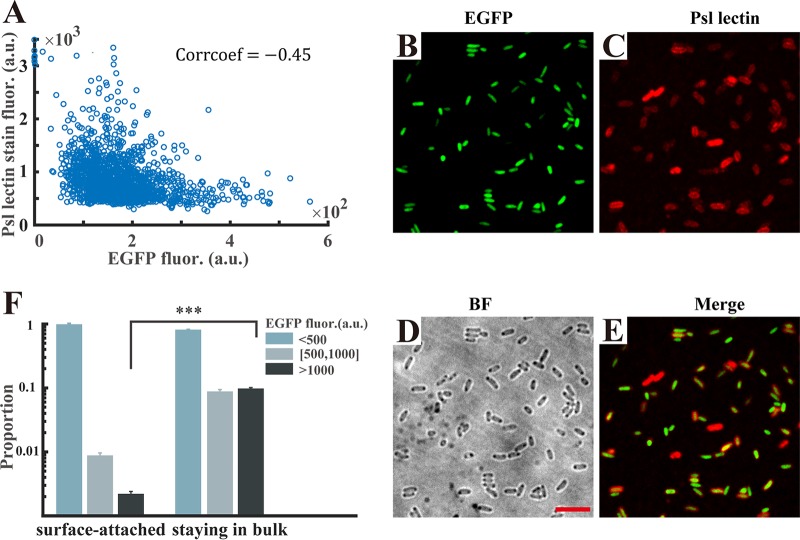
Cells that remain in the bulk have lower Psl expression than surface-attached cells. (A) The inverted reporter functionality was validated using Psl lectin staining: the correlation coefficient between the EGFP intensities and the fluorescent intensities caused by lectin staining is negative (−0.45). Every blue circle in the plot diagram represents one counted cell; more than 10^3^ cells from 3 replicates were analyzed. (B) Representative fluorescence microscopy of P. aeruginosa cells harboring the inverted Psl expression reporter. (C) Corresponding Psl lectin-stained fluorescence image. (D) Bright-field (BF) image showing all cells. (E) An overlay of EGFP reporter (B) and lectin-stained (C) fluorescence images is depicted. Scale bars for all images are 5 μm. (F) The cells staying in the bulk after attaching to a glass surface have a 10-fold-higher proportion of cells with lower Psl expression (fluorescence intensity of EGFP greater than 500 a.u.) than surface-attached cells (20% versus 2%), implying that the slow-attaching phenotype cells have a lower Psl expression level. Error bars represent means SDs with three biological replicates. ***, *P* < 0.001 (Student's *t* test).

We next intended to employ a gene expression reporter to examine the difference in the expression of Psl in single planktonic cells of P. aeruginosa. The *psl* operon is composed of 16 genes (*pslABCDEFGHIJKLOMNP*) that govern the synthesis and secretion of Psl (39, 40). We first constructed several direct reporters for the detection of Psl expression in which the intact upstream regions of the *psl* operon were directly fused to a fluorescent protein, but the fluorescence signals of the direct reporters were not sufficiently strong to be observed (Fig. S6). Karig and Weiss presented a technique for detecting weak responses using signal-amplifying genetic circuits and applied this technique to reveal previously undetectable responses of several quorum sensing controlled promoters from P. aeruginosa (41). That the technique can amplify weak signals with greater sensitivity inspired us to construct similar genetic circuits. Therefore, we finally engineered an inverted reporter to amplify the weak signal of the *psl* operon (see Materials and Methods). Particularly, in the strain harboring the inverted amplifier reporter, the enhanced green fluorescent protein (EGFP) fluorescence, as a proxy for the Psl expression level, should be negatively related to the expression of the *psl* operon. To validate the use of EGFP fluorescence for measuring the Psl production at a single-cell level, we used a tetramethyl rhodamine isocyanate (TRITC)-conjugated, Psl-specific lectin (42) to stain the cells and simultaneously measured the inverted-reporter-caused EGFP fluorescence in individual cells. We observed that the EGFP intensities were negatively related to the fluorescent intensities caused by lectin staining (correlation coefficient = −0.45 [Fig. 3A]), indicating that cells with higher levels of *psl* operon expression (lower EGFP intensities) produced more Psl (Fig. 3B to E). This result validated the functionality of the inverted reporter. Subsequently, we examined the difference in Psl expression between surface-attached bacteria and cells remaining in the bulk. Wild-type PAO1 carrying the inverted Psl expression reporter strain was used for attachment experiments. After 30 min, the surface-attached cells were immediately imaged for measuring the EGFP fluorescence intensity and the bulk populations were gently pipetted out for imaging using the agarose slab method (see Materials and Methods). We observed that the surface-attached cells showed a higher Psl expression level, with only 2% of cells having an EGFP intensity greater than 500 arbitrary units (a.u.), whereas that proportion of the cells remaining in the bulk accounted for 20%, with 10% greater than 1,000 a.u. (Fig. 3F), which indicates that the cells with the slow-attaching phenotype have less production of Psl and these cells are more likely to stay at the mobile phase. Additionally, we tracked the attached cells using the sophisticated algorithm mentioned above to exclude the possibility that the cells having higher Psl expression may be due to attachment (Fig. S7). These results demonstrate that the planktonic cells have differential production of Psl, which enables planktonic P. aeruginosa to differently attach to surfaces.

### The RsmYZ/RsmA pathway mainly regulates the differentially expressed *psl* operon in P. aeruginosa.

**FIG 4:**
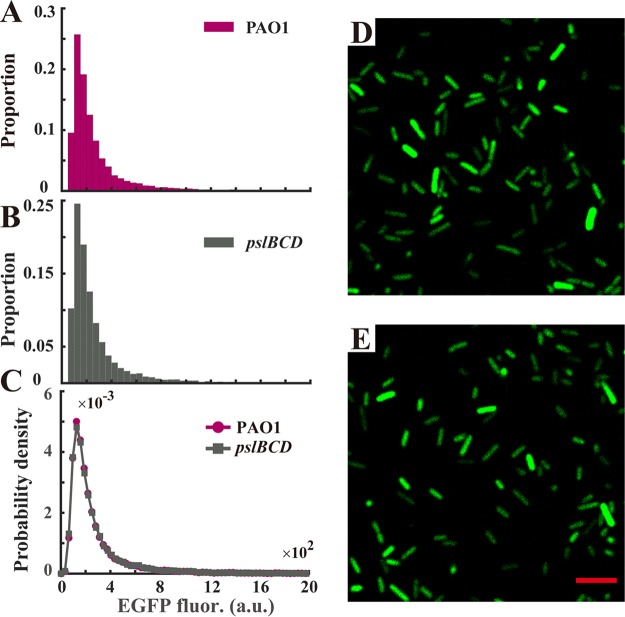
Differentially expressing *psl* operon is not caused by auto-induction of its expression of extracellular polysaccharide Psl. (A and B) Distribution of EGFP fluorescence of single planktonic cells for PAO1 (A) and PAOYs1 (Δ*pslBCD* mutant) (B) harboring the inverted Psl expression reporter in the exponential phase (cell number, >10^4^, collected from 3 replicates). (C) Probability density estimate at 100 points for the fluorescence intensity of single cells. The estimate is based on a normal kernel function, and the results show that almost the same distributions were observed between the two strains, indicating that phenotypic variability still existed in PAOYs1. (D and E) Fluorescence images were obtained by confocal microscopy of the inverted Psl expression reporter for the strains PAO1 (D) and PAOYs1 (E), respectively. Scale bars, 5 μm.

Autoinduction is a common mechanism that enables single cells to differentially express genes with epigenetic traits (43). To examine whether the differentially expressed *psl* operon in single planktonic cells is attributed to autoinduction (44), we knocked out the genes *pslBCD* in the Psl reporter strain. Figure 4 shows that almost the same distributions of fluorescence were observed for the strain PAO1 and the strain PAOYs1 (Δ*pslBCD* mutant) (PAO1, mean of 3.12 × 10^2^ a.u. and standard deviation of 3.08 × 10^2^ a.u.; PAOYs1, mean of 3.23 × 10^2^ a.u. and standard deviation of 3.14 × 10^2^ a.u.), indicating that the mutant cells (Δ*pslBCD*) still differentially expressed the *psl* operon. The results imply that the differentially expressed *psl* operon results from upstream regulations. Subsequently, we knocked out the transcriptional regulators of *algU*, *rpoS*, *ppyR*, *lasR*, and *amrZ* or the posttranscriptional regulator of *rsmA* in the wild-type strain; these factors have been shown to regulate the expression of the *psl* operon (35, 45–49). Our results indicated that the planktonic cells of the strain PAOYs11 (Δ*rsmA* mutant) uniformly attached to the surface (Fig. 2Axii), whereas the planktonic cells of strains PAOYs6, PAOYs7, PAOYs8, PAOYs9, and PAOYs10 still differently attached to the surface (Fig. 2Avii to xi, B, and C). RsmA is a global regulator (34, 50) that directly or indirectly controls the expression of hundreds of genes in P. aeruginosa through a small RNA regulational pathway (RsmYZ/RsmA). Consequently, knocking out *rsmA* may affect global genes expressions in P. aeruginosa. To further confirm that the RsmYZ/RsmA pathway mainly regulates the differentially expressed *psl* operon in the planktonic cells of P. aeruginosa, on the one hand, we replaced the regulation motif overlapping the genuine ribosome binding site (RBS) in the *psl* operon with a designed constitutive prokaryotic RBS, BBa_B0034 (iGEM biobrick), which cannot be regulated by RsmA; on the other hand, we constructed an Δ*rsmA* Δ*pslBCD* double mutant strain, PAOYs12. It was shown that after the replacement of the RBS in the *psl* operon (strain B0034*psl*-PAO1), the planktonic cells uniformly attached to the surfaces similar to that observed in the cells of PAOYs11, whereas the planktonic cells of the PAOYs12 mutant showed the slow-attaching phenotype, similar to the cells of the strain PAOYs1 (Δ*pslBCD* single mutant), indicating that the fast-attaching phenotype of the Δ*rsmA* mutant is caused by its direct regulation of the *psl* operon (Fig. 2Axiii and xiv, B, and C).

**FIG 5:**
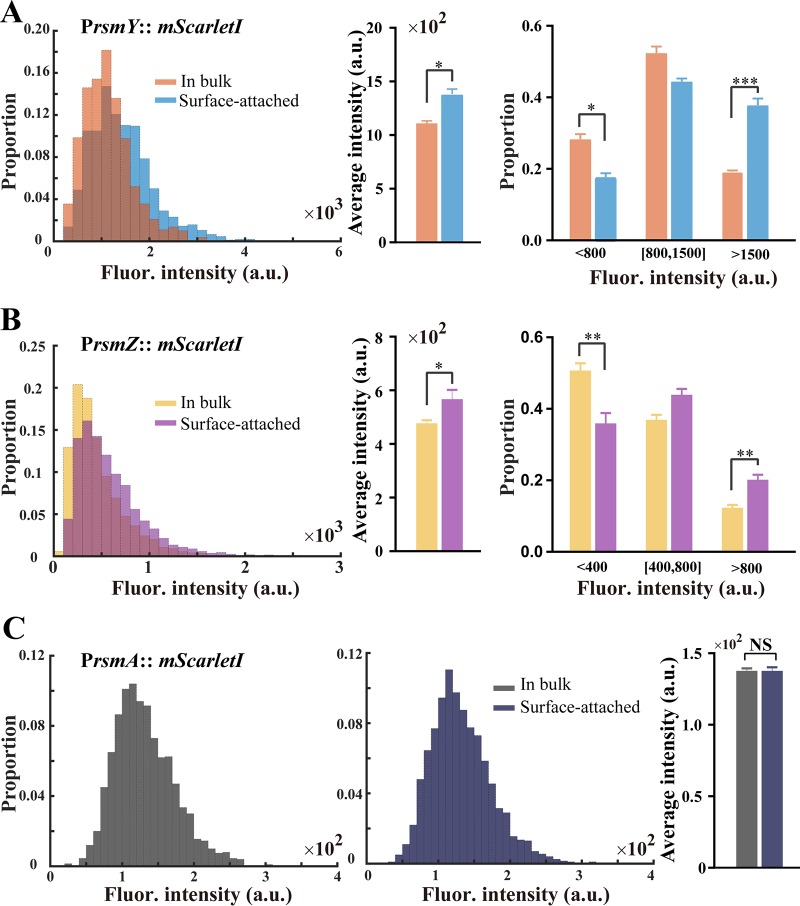
RsmYZ/RsmA regulates the differentially expressed *psl* operon in P. aeruginoasa. The RsmY, RsmZ, and RsmA expression levels were determined between the populations of bulk and surface-attached cells by measuring the fluorescence of the corresponding reporter. (A) Difference in expression of RsmY between two populations. (B) The difference of RsmZ expression was shown in a similar way. (Left) Distribution of single cell fluorescence for two populations (cell number, >3,000, collected from 3 replicates); (middle) mean fluorescence intensity; (right) analytical classification results. The results show that the proportion of small RNAs (fluorescence intensities smaller than 800 a.u. and 400 a.u. for RsmY and RsmZ, respectively) for bulk populations was approximately two times as high as for surface-attached cells (RsmY, 28% versus 17%, and RsmZ, 50% versus 35%), whereas the surface-attached cells had approximately a 2-fold-higher proportion of small RNAs (fluorescence intensities larger than 1,500 a.u. and 800 a.u. for RsmY and RsmZ, respectively) than bulk populations (RsmY, 37% versus 19%, and RsmZ, 20% versus 12%), which indicates that the cells in bulk had a lower expression of RsmY and RsmZ. (C) The distribution of RsmA levels for bulk (left) and surface-attached (middle) cells and the mean intensity (right) are displayed. Nearly the same distributions were observed and no differences were detected in RsmA expression between the two populations. In panels A, B, and C, error bars represent SDs from three biological replicates. NS, not significant. *, *P* < 0.05; **, *P* < 0.01; ***, *P* < 0.001 (Student's *t* test).

Next, we aimed to determine the RsmY/RsmZ/RsmA levels in bulk and surface-attached cells. As small RNAs' functionalities are manifested at a transcriptional level, we constructed transcriptional (*rsmY* and *rsmZ*) or translational (*rsmA*) fluorescence reporter fusions to these genes, respectively. The transcriptional reporter of *rsmY* and *rsmZ* carries an RNase III processing site (51) that is located between the promoter and the RBS of the *mScarletI* gene (52), which ensures construction of transcriptional fusions that are translated independently of the fusion sequences, and we engineered direct translational fusions of *rsmA* to the *mScarletI* gene. In a way similar to that used for measuring Psl expression difference between the bulk and surface-attached cells, we determined the RsmY, RsmZ, and RsmA levels for the two populations by measuring the mScarletI fluorescence of the corresponding reporter. Our results show that the level of RsmY expression in surface-attached cells was slightly higher than in bulk populations, and similar results were obtained for RsmZ expression (Fig. 5A and B; left and middle). Further analytical results demonstrated that the proportion of low small RNAs (fluorescence intensities less than 800 a.u. and 400 a.u. for RsmY and RsmZ, respectively.) for bulk populations was approximately two times as high as for surface-attached cells (Fig. 5A and B; right; RsmY, 28% versus 17%, and RsmZ, 50% versus 35%), whereas the proportions of high small RNAs were much lower (RsmY, 19% versus 37%, and RsmZ, 12% versus 20%). Additionally, we did not detect any difference in RsmA expression between the two populations (Fig. 5C). Together, our results demonstrated that the RsmYZ/RsmA pathway mainly regulates the differentially expressed *psl* operon in planktonic cells of P. aeruginosa.

**FIG 6:**
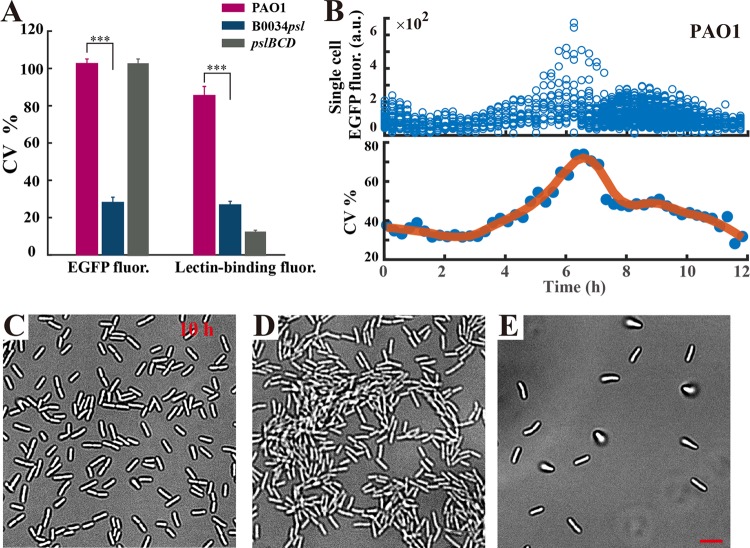
Differential production of Psl impacts cell behavior during biofilm development in P. aeruginosa. (A) The expression heterogeneity of the *psl* operon was quantified by computing the coefficient of variation (CV) of EGFP fluorescence (left), and the heterogeneity of Psl expression was determined by computing the CV of lectin binding fluorescence for PAO1, B0034*psl*-PAO1, and PAOYs1 (Δ*pslBCD* mutant) (right). Error bars correspond to SDs from three replicates. ***, *P* < 0.001 (Student's *t* test). (B) The time-varying fluorescence intensity of wild-type cells harboring the inverted Psl expression reporter in flow cell experiments indicates that attached cells having more Psl expression (EGFP intensity lower than 300 a.u.) could divide and differentiate into cells with lower Psl expression levels (EGFP intensity greater than 500 a.u.), indicated by the CV increasing markedly (from 37% [0 h] to 78% [6 h]). (C to E) Representative bright-field images for PAO1, B0034*psl*-PAO1, and PAOYs1 in flow cells at ∼10 h. Experiments shown in panels B to E were carried out in triplicate, and representative data are shown. Scale bars, 5 μm.

We further examined the phenotypic difference in expression of the *psl* operon among strains PAO1, B0034*psl*-PAO1, and PAOYs1. A corresponding inverted reporter for strain B0034*psl*-PAO1 was constructed in which the regulation motif was similarly replaced with B0034. We then quantified the expression heterogeneity of the *psl* operon by computing the coefficient of variation (CV; standard deviation divided by the mean) (53) as the relative deviation of EGFP fluorescence. Indeed, a small CV corresponds to a tight distribution centered around the mean, hence a small cell-to-cell variability; a large CV corresponds to a loose distribution, indicating large cell-to-cell variability. The results show that PAO1 and PAOYs1 have a 3-fold-higher CV for EGFP fluorescence than B0034*psl*-PAO1, indicating a differentially expressed *psl* operon in PAO1 and the PAOYs1 mutant (Fig. 6A, left part). Although PAOYs1 shows only the slow-attaching phenotype because of deficiency in production of Psl, the regulation motifs of the *psl* operon still function, and thus, the expression of the *psl* operon remains differential. Likewise, we quantified the Psl expression by computing the CV of lectin binding fluorescence. The cells of each strain were stained by the Psl-specific lectin and the binding fluorescence was measured in individual cells. The CV for lectin binding fluorescence for PAO1 was approximately three times as high as that for the strain B0034*psl*-PAO1 and much higher than that for PAOYs1, consistent with our previous results showing that the levels of expression of Psl of PAOYs1 and B0034*psl*-PAO1 are more uniform (Fig. 6A, right part). Collectively, these results further confirmed that planktonic cells of P. aeruginosa produced polysaccharide Psl differentially and the RsmA pathway mainly regulates the differentially expressed *psl* operon.

### Differential production of Psl in planktonic cells impacts biofilm formation.

**FIG 7:**
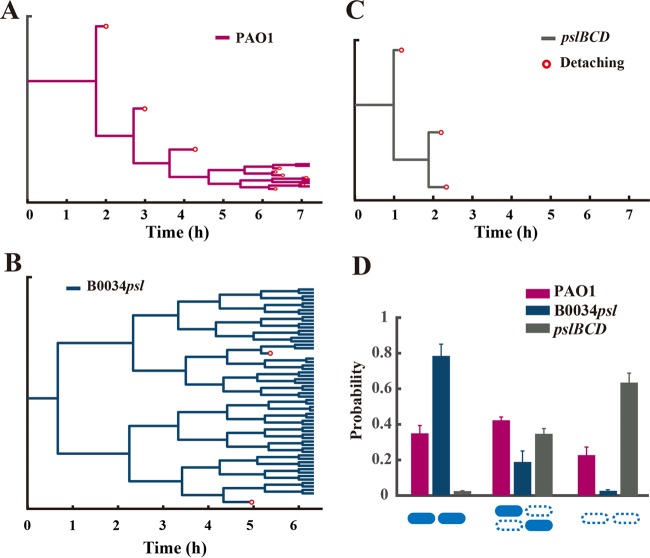
Differential production of Psl affects postdivision cell fates during biofilm development in a flow cell system. (A to C) Shown are reconstructions of the genealogical trees from one mother cell in flow cell experiments for strain PAO1 (A), strain B0034*psl*-PAO1 (B), and strain PAOYs1 (Δ*pslBCD* mutant) (C). Red circles represent detaching events. (D) Probability of postdivision cell fates; filled rods indicate staying and open dashed rods indicate leaving Error bars are estimated from $1/\sqrt{N_{\text{div}}}$, where *N*_div_ is the total number of division events from at least three independent experiments during the period of interest (*N*_div_ > 200). The results show that Psl differentially expressing strain PAO1 exhibits asymmetrical detaching behaviors after divisions, whereas the two daughter cells of strain B0034*psl*-PAO1 or PAOYs1 both prefer to attach on the surface or detach from surface, respectively.

To investigate whether the differential production of Psl in planktonic cells affects subsequent biofilm formation, we continuously cultured the Psl differentially expressing (wild-type) strain, the Psl uniformly expressing strain (B0034*psl*-PAO1), and the slow-attaching strain PAOYs1 in a flow cell, thereby enabling these strains to form biofilms. First, using bright-field microscopy, we carefully determined the difference in biofilm formation in the early stage among these three strains. Subsequently, we used a high-throughput bacterial tracking algorithm to identify division and postdivision cell fates (54) and further reconstruct the genealogical trees from one mother cell (Fig. 7A, B, and C). We distinguished three possibilities: both postdivision cells attach or leave or one cell stays and the other leaves (see “Data analysis” in the supplemental material). The probability of only one cell staying accounts for the largest proportion for the wild type (higher than 40% [Fig. 7D]). In contrast, the probability of both cells staying for the B0034*psl*-PAO1 mutant approaches 80%, approximately three times higher than that for the wild type, and the probability of both cells leaving for strain PAOYs1 has the highest proportion (60% [Fig. 7D]). Our results indicated the following. (i) The two daughter cells of the Psl differentially expressing strain PAO1 typically exhibited asymmetrical detachment behaviors after division (Fig. 7A); one daughter cell preferred to remain attached on surfaces, whereas the other preferred to detach from surfaces (Fig. 7A and D). (ii) In contrast, the daughter cells of the Psl uniformly expressing strain B0034*psl*-PAO1 both preferred to remain attached on surfaces (Fig. 7B and D), and both daughter cells of PAOYs1 were prone to detach under flow as a result of deficient production of Psl (Fig. 7C and D). These findings suggest that the differential production of Psl in single cells enables one daughter cell to detach from the surface during the early stage of biofilm formation, presumably because the cells producing less Psl preferred to detach. Most interestingly, by monitoring PAO1 cells harboring the inverted Psl expression reporter in the flow cell experiment, we found that the initially attached cells with higher Psl expression (EGFP intensity lower than 300 a.u.) could divide and differentiate into cells with lower Psl expression (EGFP intensity greater than 500 a.u.), indicated by the CV of EGFP fluorescence increasing markedly from 37% (0 h) to 78% (Fig. 6B, 6 h). In contrast, there are only small fluctuations around a smaller (35%) or larger (110%) CV for B0034*psl*-PAO1 and PAOYs1, respectively (Fig. S8).

**FIG 8:**
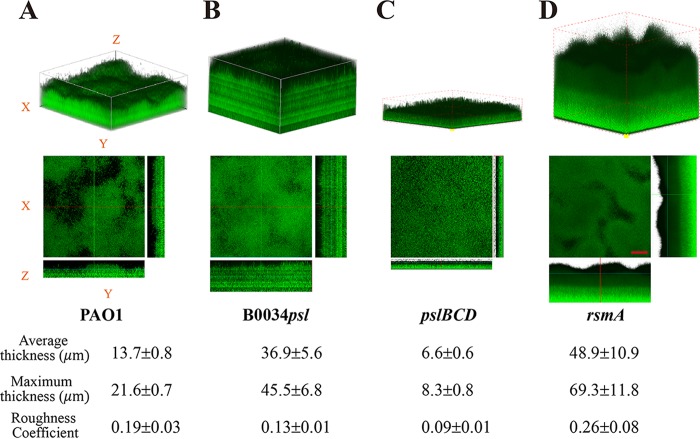
The biofilm matrix of P. aeruginosa PAO1 and its uniformly expressing Psl mutant strain B0034*psl*-PAO1. (A) Three-dimensional (3D) structures of biofilms for PAO1. (B) 3D structures of biofilms for B0034*psl*-PAO1. Biofilms for strains PAOYs1 (Δ*pslBCD* mutant) (C) and PAOYs11 (Δ*rsmA* mutant) (D) were observed as well. All strains were fluorescently tagged for visualization. The images were acquired by confocal microscopy after 4 days of cultivation. For each strain, the experiment was done in triplicate and five image stacks were obtained from five areas covering 1,270 μm by 1,270 μm along the flow cell in each experiment. A representative biofilm image for each sample is shown. The reconstructed biofilm architecture shows that biofilms of strain B0034*psl*-PAO1 are much thicker but have less roughness than that of PAO1. The biofilm thickness for PAOYs1 was only half of that for the wild type, whereas PAOYs11 formed thicker biofilms. The scale bars for the *x-y* view are 200 μm. A COMSTAT analysis of the data from each strain is shown at the bottom. The averages and standard deviations were calculated from 10 samples.

Subsequently, we found that after 10 h of cultivation in flow cell system, the Psl uniformly expressing strain B0034*psl*-PAO1 formed microcolonies (Fig. 6C) earlier than the Psl differentially expressing strain PAO1 (Fig. 6D), whereas the cells on the surface for strain PAOYs1 were still sparse at this point (Fig. 6E). Using confocal microscopy, we further examined the morphology of biofilms formed from the Psl differentially or uniformly expressing strain in the mature stage. Figure 8 shows that biofilms formed by the Psl uniformly expressing strain B0034*psl*-PAO1 were much thicker (twice the thickness of those formed by PAO1) but less rough, whereas the Psl differentially expressing strain PAO1 formed relatively thin, differentiated, and rougher biofilms. These data were reproducible and statistically significant (*P* < 0.001 for average thickness; *P* < 0.01 for roughness coefficient). We also observed the biofilms for strains PAOYs1 and PAOYs11 (Fig. 8C and D). Biofilm development for PAOYs1 was severely compromised, as the cells were deficient in production of Psl. The biofilm thickness was only one-third of that for the wild type, which is in agreement with the previous study showing that Psl is necessary to maintain normal biofilm structure (55). In contrast, the biofilms of the Δ*rsmA* strain had an average thickness higher than that of PAO1 biofilms, which may have resulted from the global effect of RsmA. The regulator RsmA affects more than 500 genes in P. aeruginosa, including type IV pili, *psl*, *pel*, alginate, the type III secretion system (T3SS), and T6SS (34), each of which has an influence on biofilm development. Taken together, our results show that the differential production of Psl in P. aeruginosa plays a significant role in various stages of biofilm formation. For instance, the differential production of Psl (i) enables subpopulations of planktonic cells to quickly attach to surfaces in the initial stage, (ii) enables subpopulations of surface-associated cells to differentiate into ones that can detach from surfaces in the early stage, and (iii) subsequently enables young biofilms to form matrix-sophisticated structures in the mature stage.

Although the bacteria within a population may be genetically identical, heterogeneity is intrinsic to individual cells. We observed that in a uniform environment, planktonic cells of P. aeruginosa differently attached to a surface even when they remained in the exponential phase and the stationary phase contained more planktonic cells with the fast-attaching phenotype. When cells enter the stationary phase, they start to suffer from the limitations of nutrition. More fast-attaching cells can help planktonic cells quickly attach to and occupy surfaces, which can accelerate the development of biofilms and further affords protection for the cells from a wide range of environmental challenges. Furthermore, in our context, we expect that such a phenotypic variation greatly affects the initial stage of biofilm formation in the presence of flows because fast-attaching cells are more likely to attach on the surface to form microcolonies, whereas slow-attaching cells are more likely to remain in the mobile phase. This scenario is different from the previous understanding of biofilm formation in the initial stage, in which planktonic cells were believed to uniformly attach on surfaces. Most notably, the results of this study show that it is the RsmYZ/RsmA regulational pathway that enables planktonic cells to differently attach to surfaces. Thus, we speculate that different approaches used by planktonic cells to attach on surfaces constitute a bet-hedging strategy that has naturally evolved in P. aeruginosa (56). The strategy enables only a subset of the population to attach on the surface, which might be or might not be a suitable habitat for biofilm formation; however, the remainder of the population can live in the mobile phase regardless of whether the attaching cells succeed in forming biofilms. This strategy is expected to provide evolutionary benefits to P. aeruginosa in overcoming unpredictable environmental perturbations (57).

## MATERIALS AND METHODS

### Strains and growth conditions.

For routine culture, P. aeruginosa were grown on LB agar plates at 37°C for 24 h. Monoclonal colonies were inoculated and cultured with a minimal medium (FAB) (58) at 37°C with 30 mM glutamate as a carbon source under aerobic conditions, in which the medium contained the following per liter of H_2_O: 2 g of (NH_4_)_2_SO_4_, 12.02 g of Na_2_HPO_4_·12H_2_O, 3 g of KH_2_PO_4_, 3 g of NaCl, 93 mg of MgCl_2_, 14 mg of CaCl_2_·2H_2_O, and 1 ml of trace metal solution. The trace metals solution contained CaSO_4_·2H_2_O (200 mg liter^−1^), FeSO_4_·7H_2_O (200 mg liter^−1^), MnSO_4_·H_2_O (20 mg liter^−1^), CuSO_4_·5H_2_O (20 mg liter^−1^), ZnSO_4_·7H_2_O (20 mg liter^−1^), NaMoO_4_·H_2_O (10 mg liter^−1^), and H_3_BO_3_ (5 mg liter^−1^). The strains not specified were harvested at an OD_600_ of approximately 2.0, and the bacterial cultures were further diluted (1:100) in fresh FAB medium to culture to the later exponential phase (OD_600_ **∼** 1.2) before use.

Bacterial strains and plasmids used in this study are listed in Tables 1, 2, and 3. Standard molecular cloning techniques were used for construction of related plasmids in Escherichia coli strain Top10. When required, antibiotics were added to medium at the following concentrations (in micrograms per milliliter): gentamicin, 15; ampicillin, 100 (E. coli); gentamicin, 30, and ampicillin, 300 (P. aeruginosa).

**TABLE 2:** Plasmids used in this study

Plasmid	Description or relevant characteristics^*a*^	Source
pUC18T-mini-Tn7T-Gm	Mini-Tn*7* transposon vector; Ap^r^ Gm^r^ on mini-Tn*7*T; *oriT* on pUC18	H. P. Schweizer
pTNS2	T7 transposase expression vector	H. P. Schweizer
pFlp2	FRT cassette vector for Flp recombinase; Ap^r^	H. P. Schweizer
pEX18Gm	Allelic-exchange vector with MCS from pUC18; *oriT sacB lacZ*α; Gm^r^	H. P. Schweizer
pEX18Ap	Allelic-exchange vector with MCS from pUC18; *oriT sacB lacZ*α; Ap^r^	H. P. Schweizer
pFGM1	Source of gentamicin FRT-*aacC1*-FRT cassette; Ap^r^ Gm^r^	H. P. Schweizer
pFTC1	Source of gentamicin FRT-*tetR*-FRT cassette; Tc^r^ Gm^r^	H. P. Schweizer
pEX18Gm_*pslBCD*	pEX18G-derived allelic-exchange vector for *pslBCD*; Gm^r^	Present study
pEX18Gm_*pilA*	pEX18Gm-derived allelic-exchange vector for *pilA*; Gm^r^	Present study
pEX18Gm_*algU*	pEX18Gm-derived allelic-exchange vector for *algU*; Gm^r^	Present study
pEX18Gm_*ppyR*	pEX18Gm-derived allelic-exchange vector for *ppyR*; Gm^r^	Present study
pEX18Gm_*rpoS*	pEX18Gm-derived allelic-exchange vector for *rpoS*; Gm^r^	Present study
pEX18Gm_*amrZ*	pEX18Gm-derived allelic-exchange vector for *amrZ*; Gm^r^	Present study
pEX18Gm_*rsmA*	pEX18Gm-derived allelic-exchange vector for *rsmA*; Gm^r^	Present study
pEX18Gm_B0034*psl*	pEX18Gm-derived allelic-exchange vector bearing BBa_B0034 substitution construct for *psl* operon; Gm^r^	Present study
RYS1	Mini-Tn*7* transposon with *egfp* directly driven by the *psl* promoter; P*psl-egfp*-T-Tn*7*; Ap^r^ Gm^r^	Present study
RYS2	Inverted Psl-expression reporter vector based on mini-Tn*7*; P*psl-lacI*-T-P*_A1/O4/O3_-egfp*-T-Tn*7*; Ap^r^ Gm^r^	Present study
RYS3	The regulation motif of *psl* operon by RsmA in RYS2 was replaced with B0034	Present study
RYS-4:P*rsmY-mScarletI*	RsmY transcriptional reporter *mScarletI* driven by the *rsmY* promoter cloned into pJN105; Gm^r^	Present study
RYS-5:P*rsmZ-mScarletI*	RsmZ transcriptional reporter *mScarletI* driven by the *rsmZ* promoter cloned into pJN105; Gm^r^	Present study
RYS-6:P*rsmA-mScarletI*	RsmA translational reporter *mScarletI* driven by the *rsmA* promoter cloned into pJN105; Gm^r^	Present study
P*_A1/O4/O3_-egfp*-T-Tn*7*	Mini-Tn*7* transposon with *egfp* driven by the P*_A1/O3/O4_* promoter; Ap^r^ Gm^r^	Present study

a Ap, ampicillin; Gm, gentamicin.

**TABLE 3:**
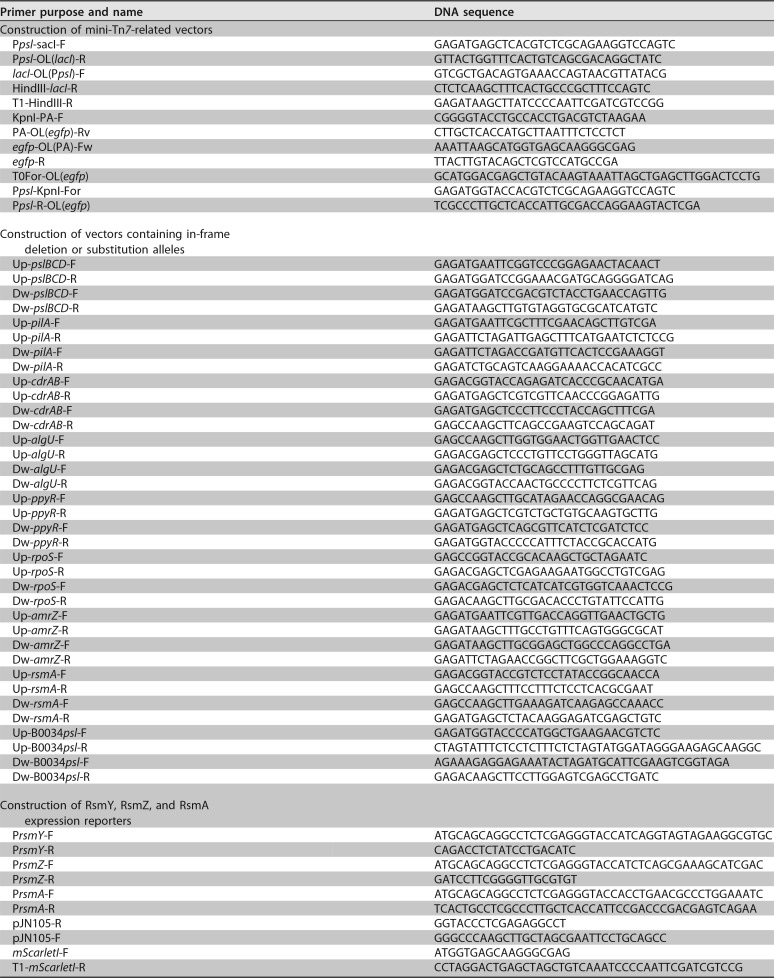
Primers used in this study

The mini-Tn*7* (59) site-specific transposition system was chosen for construction of the Psl expression reporter. The construction designed for direct detection contains *egfp* under the control of the *psl* operon promoter. Briefly, PCR fragments, promoter P*psl*, output gene *egfp*, and terminators (T0T1) were assembled into one piece, P*psl-egfp*-T, using splicing by overhang extension PCR (SOE PCR) (60), and inserted into pUC18-mini-Tn*7*-Gm (61) using the digestion-ligation method. The direct detection plasmid was named P*psl*-EGFP-T-Tn*7* for short. In a similar way, the fluorescence protein and/or vector was replaced to construct other direct reporters. The construction of the inverted Psl expression reporter was similar to that reported previously (41). The plasmid contains the P*psl-lacI*-T module, which is the part of direct detection plasmid with *egfp* replaced by *lacI*, along with *egfp* fused to the LacI-regulated P_*A1/O3/O4*_ promoter (62), named P*psl-lacI*-T-P_*A1/O4/O3*_-*egfp*-T-Tn*7*. Then the mini-Tn*7* transpositions were achieved using a rapid electroporation method (63), finally resulting in the direct reporter strain named PAO1::RYS-1 and inverted reporter strain named PAO1::RYS-2 for short. Furthermore, the selection resistance gene marker can be removed by Flp-mediated excision (64).

Δ*pslBCD*, Δ*pelB*, Δ*pilA*, Δ*cdrAB*, Δ*algU*, Δ*rpoS*, Δ*ppyR*, Δ*amrZ*, Δ*rsmA*, and Δ*pslBCD* Δ*rsmA* allelic deletion strains were constructed using well-established protocols based on two-step allelic exchange (65). Δ*algD* and Δ*lasR* strains were kindly shared by the lab of Joshua D. Shrout (University of Notre Dame) (66). The *psl* operon RBS substitution strain B0034*psl*-PAO1 was constructed using the same protocols.

The procedures for construction of small RNA transcriptional and RsmA translational reporters were similar. Taking RsmY reporter as an example, Gibson assembly (67) was used to join promoter P*rsmY*, output gene *mScarletI*, and terminators (T0T1) modules together to implement integrated RsmY reporter plasmid in single-step reactions. Briefly, four DNA fragments, including PrsmY, mScarletI, T0T1, and linearized vector pJN105, that overlapped in sequence by ∼25 bases were constructed by PCR through the design of PCR primers that contain overhangs, which provide sequence overlap with adjacent fragments. Next, 100 ng of the linearized vector backbone pJN105 and equimolar amounts of the other assembly pieces were added to 10 μl of NEBuilder HiFi DNA assembly master mix in a 20-μl total volume assembly reaction mixture. The assembly reaction mixture was incubated at 50°C for 60 min, and then 5 μl of the assembly reaction was transformed into 100 μl of competent E. coli. Finally, the reporter plasmid P*rsmY-mScarletI*-T-pJN105 was confirmed by sequencing.

### Mathematical model.

As mentioned above, our experimental condition enabled the total number of cells [*N* = *N_s_*(*t*) +*N_b_*(*t*)] to remain constant. We assumed that *i* distinctive phenotypes (*i* ≥ 1) coexisted in the planktonic cells. Subsequently, we used a rate equation to describe the kinetics of attachment of planktonic cells to surfaces, expressed as follows:

(1) $$\frac{dN_{b}(t)}{dt}=-\frac{dN_{s}(t)}{dt}=\sum_{i}\alpha_{i}N_{b}^{i}(t)-\sum_{i}\beta_{i}N_{s}^{i}(t)$$

where α_*i*_ or β_*i*_ represents the attaching or detaching rate of the *i*th phenotype, and $N_{b}^{i}$ or $N_{s}^{i}$ represents the cell numbers in the bulk or on the surface of the *i*th phenotype, $N_{b}(t)=\Sigma_{i}N_{b}^{i}(t)$ or $N_{s}(t)=\Sigma_{i}N_{s}^{i}(t)$, respectively. We observed that the number of the net attaching cells $\Sigma_{i}\alpha_{i}N_{b}^{i}(t)$ was much higher than that of the net detaching cells $\Sigma_{i}\beta_{i}N_{s}^{i}(t)$ (Fig. S4), and this enabled us to neglect the detaching term $\Sigma_{i}\beta_{i}N_{s}^{i}(t)$ in equation 1. Based on this simplification, we solved equation 1 by using an initial condition, *N_b_*(0) = *N*. The solution to equation 1 is expressed as follows:

(2) $$\frac{N_{b}(t)}{N}=\sum_{i}\frac{N_{b}^{i}(0)}{N}\exp(-\alpha_{i}t)$$

In our study, the attaching kinetics of the planktonic cells for all strains were described by the above equation. Other information and descriptions helpful for understanding could be found in the supplemental material (“Data analysis” and Fig. S9).

### Attachment experiments.

Diluted bacterial culture was incubated in shaker with 250 rpm at 37°C and collected by centrifugation at different growth phases indicated by OD_600_. The collected cells were washed twice with FAB medium without addition of a carbon source to ensure that the number of cells, either in bulk or on the surface, was not influenced by uncertain cell divisions. The resultant cells were further diluted by adding the proper volume to 1 ml of fresh FAB to an OD_600_ of ∼0.02 for use. A cover glass-bottom dish was prepared in advance for providing windows to image and then placed into the incubator of microscope with right focus locked. After gentle addition of the suspended cells, the glass surface of the dish was monitored with a camera, with bright-field images (165 μm by 139 μm) recorded every 3 s for a total time of about 1 h at 30°C. Cells of each P. aeruginosa mutant strain for attachment experiments were harvested at the identical growth phase (later exponential phase, OD_600_ of ∼1.2). Each mutation had no discernible effects on cell growth (Fig. S5).

After acquisition of bright-field images, the 16-bit greyscale images were first converted to binary images for the detection of bacteria with a standard image processing algorithm that was coded in MATLAB, and the *x-y* positions of leading and trailing poles in single cells were then determined and linked individually over time by using our established two-point tracking algorithm. More detailed information has been provided previously (32, 33). In each attachment experiment, more than 300 cells were tracked for analyzing the attaching kinetics. For Fig. 1 and 2, attachment experiments were all carried out at least three times and one representative example is shown.

### Agarose slab experiments.

P. aeruginosa wild-type PAO1 carrying the inverted Psl expression reporter strain was used for attachment experiments as descried above. The surface-attached cells were immediately imaged using a confocal microscope for measuring the EGFP fluorescence intensity of single cells. The cells in bulk were gently pipetted out and then loaded on top of an agarose slab made of 2% (wt/vol) agarose with FAB medium. The pipetted bacterial solution needs minutes to evaporate and absorb into the agarose for ensuring that all bulk cells land at the slab. After flipping the agarose pads onto a cover glass-bottom dish, with the bacteria sandwiched between the agarose slab and the cover glass, finally the EGFP fluorescence intensities of single bulk cells were acquired by confocal imaging. Similar methods were used for measuring the difference in RsmY, RsmZ, and RsmA expression between two populations of surface-attached and bulk cells.

To determine whether the number of resuspended cells for attachment experiments was constant, we checked the division events and growth rate under the condition of the absence of a carbon source. The resuspended cells were loaded on top of an agarose slab, and then the slab was flipped and transferred to an imaging dish. Bright-field images were acquired to monitor the behaviors of cells for 1 h at 30°C to give the same circumstances as in the attachmen experiments. Cells loaded on the FAB agarose slab containing 30 mM sodium glutamate acted as a control group. We used a combination of a high-throughput bacterial tracking algorithm (see “Data analysis” in the supplemental material) and manual validation to analyze the images.

### Flow cell experiments and biofilm cultivation.

Biofilms were grown at room temperature in flow cells, which were purchased from the Department of Systems Biology, Technical University of Denmark, and assembled using a standard protocol (58, 68). Diluted bacterial cultures were injected into flow cells and left inverted for 15 min to allow attachment of cells to the coverslip. After unattached cells were washed out, surface-attached cells were subsequently cultured by flowing FAB medium with 0.6 mM glutamate at a constant flow rate (3.0 ml h^−1^). In the first 20 h, the behaviors of single cells were continuously monitored using bright-field images. Afterwards, the flow cells containing the young biofilms were continuously cultured for up to 4 days before biofilm image acquisition, which allowed the formation of mature biofilms. Each experiment was performed in triplicate, and 10 positions were analyzed for calculating the averages and standard deviations. Captured images of biofilms were subjected to quantitative image analysis for thickness and roughness using COMSTAT software (69).

The experiments with strains carrying the inverted Psl expression reporter were conducted with a flow cell system, but for the imaging, a spinning-disk confocal microscope was applied to record fluorescent micrographs for every 20 min. The time-varying fluorescence intensities of single cells were employed to assess the evolution of Psl expression.

### Lectin staining.

Psl was stained with 100 μg ml^−1^ of TRITC-labeled Hippeastrum hybrid lectin from amaryllis (EY Laboratories, Inc.) as previously described (42). Summarily, the cultural cells pregrown in FAB medium were collected by centrifugation and rinsed twice with phosphate-buffered saline (PBS); cells were redispersed with diluted fluorescently labeled lectin (100 μg ml^−1^) using PBS. Staining was done for 1 h in the dark at 37°C. Samples were washed three times with PBS and then imaged with a microscope. Preparations for imaging sample are described under “Agarose slab experiments” above.

### Microscopy and image acquisition.

Fluorescence imaging was performed using a spinning-disk confocal (CSU-X1; Yokogawa) inverted microscope (IX81; Olympus) equipped with a Zero Drift autofocus system incubator (model NUB-ZILCSGH-F1; Tokai Hit), laser combiner system (Andor Technology), a 100×, 1.4 numerical aperture (NA) oil immersion objective (Olympus), and an electron multiplying charge-coupled device (EMCCD) camera (iXon897; 512 by 512 pixels). The microscope, the camera, and the stage were actuated with live-cell imaging software Andor iQ. The green channel filter (488-nm exciter and 524/40-nm emitter) and red channel filter (561-nm exciter and 605/40-nm emitter) set with a dichroic beam splitter (Semrock) were used for detection of EGFP fluorescence and Psl stain fluorescence. The bright-field images for attachment experiments or flow cell experiments were recorded using an sCMOS camera (Andor Neo; 2,560 by 2,160 pixels) every 3 s for a total recording time of about 1 h or 20 h, respectively. The different z-position fluorescent images of biofilms were acquired with the 488-nm laser line from an argon laser (GLG3135; Showa Optronics) using z-axis scanning (0.6 μm per step) by confocal scanning laser microscopy (FLUOVIEW-FV1000; Olympus).

### Statistical analysis.

Images acquired were analyzed using ImageJ v.1.51w software or computed using MATLAB (MathWorks) codes as reported previously (31). To compute the average fluorescence intensity, we subtracted the average fluorescence per pixel of the background from the average intensity per pixel in the given cell and more than 10^3^ cells were analyzed to obtain the mean fluorescence intensities of bacterial populations. Mean values and standard deviations were obtained from at least two independent experiments (biological replicates). Statistical analysis was performed with Student's unpaired two-sided *t* test using GraphPad Prism version 7.03.

## Supplementary Material

Supplemental material
