# Supplementary material for: Differential Production of Psl in Planktonic Cells Leads to Two Distinctive Attachment Phenotypes in Pseudomonas aeruginosa
Source: Appl Environ Microbiol. 2018 Jul 2;84(14):e00700-18. doi: 10.1128/AEM.00700-18 (PMC6029103; doi:10.1128/AEM.00700-18)
Supplement: Supplemental material [file supp_84_14_e00700-18__index.html]

Supplemental material 

# Differential Production of Psl in Planktonic Cells Leads to Two Distinctive Attachment Phenotypes in Pseudomonas aeruginosa

## Supplemental material

- Supplemental file 1 -

  *Pseudomonas aeruginosa* growth curve (Fig. S1); evidence that division events were not detected for the cells deprived of a carbon source in the first hour (Fig. S2); determination of death rate using the PI staining method for planktonic cells (Fig. S3); time dependence of net attaching and detaching cell number in one attachment experiment (Fig. S4); evidence of lack of discernible effects of mutations on bacterial cell growth (Fig. S5); weakness of direct expression reporter signals of *psl* operon (Fig. S6); tracking of attached cells to analyze the time duration on the surface and the change in fluorescence of the inverted reporter (Fig. S7); changes in Psl expression in the process of biofilm formation in a flow cell system for PAO1, Δ*pslBCD*, and B0034*psl*-PAO1 strains (Fig. S8); mathematical model illustrating the physics image near the switch point (Fig. S9); supplemental methods: data analysis.

  PDF, 1.8M
