# Supplementary material for: Differential Production of Psl in Planktonic Cells Leads to Two Distinctive Attachment Phenotypes in Pseudomonas aeruginosa
Source: Appl Environ Microbiol. 2018 Jul 2;84(14):e00700-18. doi: 10.1128/AEM.00700-18 (PMC6029103; doi:10.1128/AEM.00700-18)
Supplement: Supplemental material [file AEM.00700-18_zam014188626s1.pdf]

Supplemental material

Differential production of Psl in planktonic cells leads to two distinctive attaching phenotypes in *Pseudomonas aeruginosa*

Shuai Yang[a], Xinyi Cheng[a], Zhenyu Jin[a], Aiguo Xia[a], Lei Ni[a], Rongrong Zhang[a] and Fan Jin[a,b,c #]

a. Hefei National Laboratory for Physical Sciences at the Microscale, University of Science and Technology of China, Hefei 230026, P. R. China

b. Department of Polymer Science and Engineering, University of Science and Technology of China, Hefei 230026, P. R. China

c. CAS Key Laboratory of Soft Matter Chemistry, University of Science and Technology of China, Hefei 230026, P. R. China

# Address correspondence to Fan Jin, fjinustc@ustc.edu.cn; Tel.: +86-551-63606925; Fax: +86-551-63606743.

S.Y and XY.C. contributed equally to this work.

Contents

Supplemental figures **3**

Figure S1 3

Figure S2 4

Figure S3 5

Figure S4 6

Figure S5 7

Figure S6 9

Figure S7 10

Figure S8 11

Figure S9 12

Supplemental Methods **12**

Data Analysis 12

Data analysis for attaching experiment 13

Data analysis for flow cell experiment 15

Data analysis for fluorescent images 16

Supplemental References **17**

Supplemental figures

Figure S1

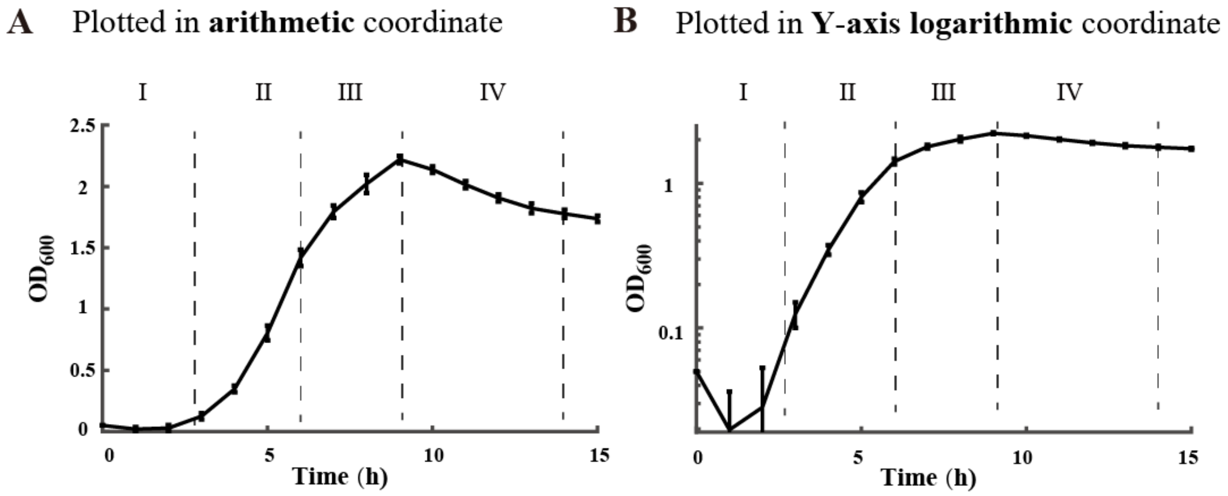

Supplemental Figure 1. *Pseudomonas aeruginosa* growth curve. The OD_{600} of PAO1 planktonic cells growing in FAB containing 30 mM glutamate was measured for 15 hours. The grow curve were presented in arithmetic coordinate (**A**) and Y-axis logarithmic coordinate (**B**). The growth kinetic curve can be modeled with four different phases: lag phase (I), exponential phase (II), stationary phase (III), and death phase (IV). We recognized that the stationary phase starts from the decrease of growth rate based on the definition by Kolter *et al* (1). Different growth phase of bacteria cells were used to carry on attaching experiments. Error bars represent means ± s. d with n=3 biological replicates.

Figure S2

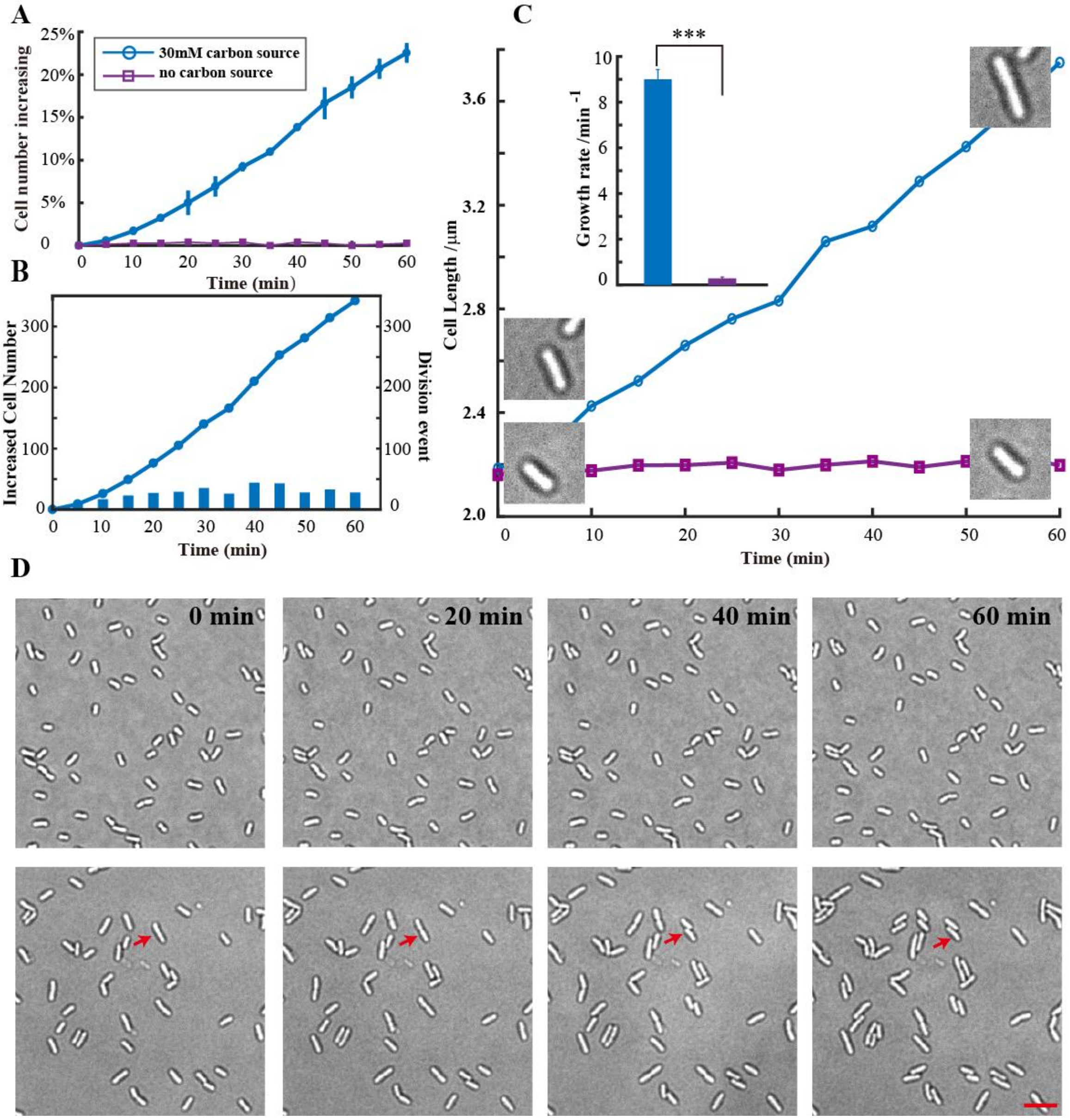

Supplemental Figure 2. Division events were not detected for the cells deprived of carbon source in the first hour. We loaded the resuspended cells on top of an agarose slab made of 2% (w/v) agarose with FAB medium with (blue circle) or without (purple square) carbon source (30 mM sodium glutamate). Slabs were then flipped and transferred to an imaging dish, and bright-field images (2560×2160 pixel) were acquired to monitor the behaviors of cells for 1 hour at 30℃ to give the same circumstances with attaching experiment. **A.** The number of cells deprived of carbon source remains

constant compared with that of cell with carbon source increased substantially by 20%. Each data set is normalized by the total number of the first frame. Error bars represent means ± s. d, each data point represents the mean values from n=3 fields. **B.** Division events were barely observed for the cells without carbon source in the first hour, but for the cells under the containing carbon source conditions, 342 division events were detected in the first hour (the initial number of cell is 1517). Time course of vertical bar graph is the net division events during every 5 minutes. **C.** Representative curves of cell-size dynamics were shown respectively. Insert, average growth rate was computed from more than 500 cells (collected from 3 replicates), error bars means ± s. d, ***$P < 0.001$ (Student's *t*-test). We found that cells hardly grow in medium without carbon source in one hour, and statistics show that the growth rate under the rich medium conditions was more than 30 times higher than under no carbon source conditions (9.0×10^{-3} $\min^{-1}$ versus 2.8×10^{-4} $\min^{-1}$). **D.** Time-lapse bright-field image (partial, 584×584 pixel) of cells under the condition of without (upper) and with carbon source (lower). The red arrows indicate a division process of one cell. Scale bar, 5 μm. The experiments were carried out two times and the depicted example is for the cells in the later exponential phase (OD_{600}~1.2).

Figure S3

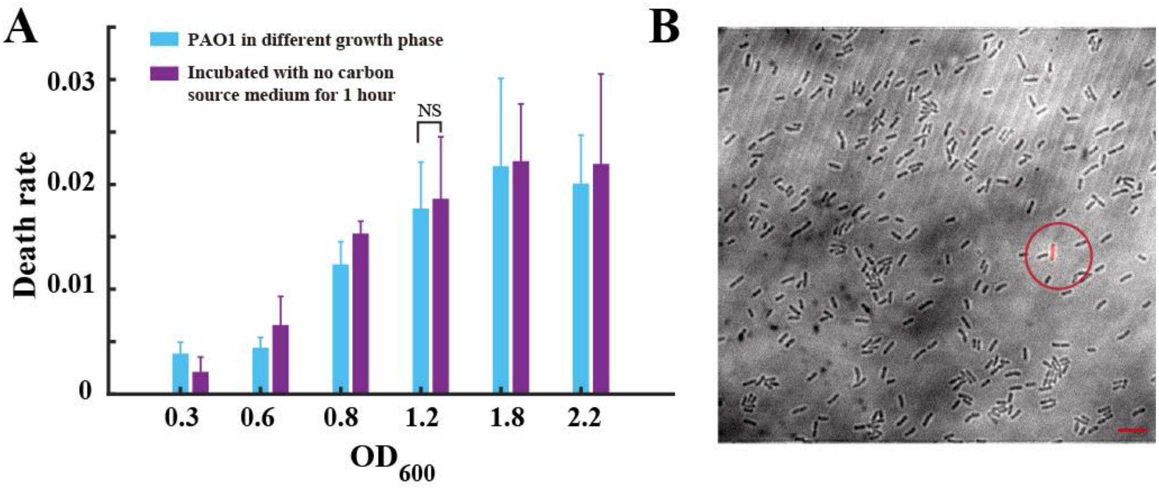

Supplemental Figure 3. Death rate were determined using PI-staining method for planktonic cells. **A.** Bar graph of the death rate for the planktonic cells in different growth phase and after they are transferred to no carbon source conditions and incubated for 1 hour. The cells were collected at

different growth phase. 100 μL of the cultural were immediately stained by PI and the rest of the culture were treated in a similar method with attaching experiments. Simply, the cells were washed by FAB medium without adding carbon source, and then re-suspended in fresh FAB and incubated for 1 hour at 30℃. After that, the cells were ready for staining. The dead cells were recognized by red fluorescence with using confocal microscope, and More than 10^4 cells were counted for analyzing death rate. We found that under adding 30mM glutamate conditions, dead cells are hardly observed before stationary growth phase, and the death rate reached to a maximum only 2 % in the late stationary phase. After the cells were incubated in no carbon source medium for 1 hour, no increase of the death rate was observed. Error bars represent means ± s.d with three biologic replicates, NS = not significant (Student's *t*-test). **B.** A representative image of overlay staining fluorescence and bright-field image for cells in the later exponential phase. Only one dead cell was detected in the field, indicated by red circle. Scale bar, 5 μm.

Figure S4

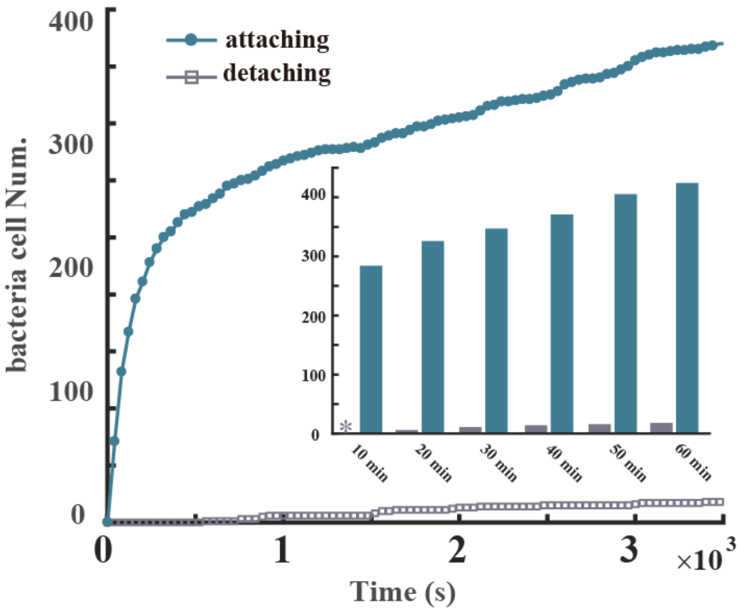

Supplemental Figure 4. Time dependence of net attaching and detaching cell number in one attaching experiment. The attaching experiment of PAO1 cultured to an OD_{600}~1.2 was used for presentation. Similar results were observed for cell in other growth phases. The number of attaching and detaching cells were counted using bacterial tracking algorithm. The detaching events occurs rarely with the number of net detaching cells having only been less than 5 percent of that of net attaching cells in 1 h. Insert, vertical bar graph of net attaching and detaching cell number at different time point for easily read.

Figure S5

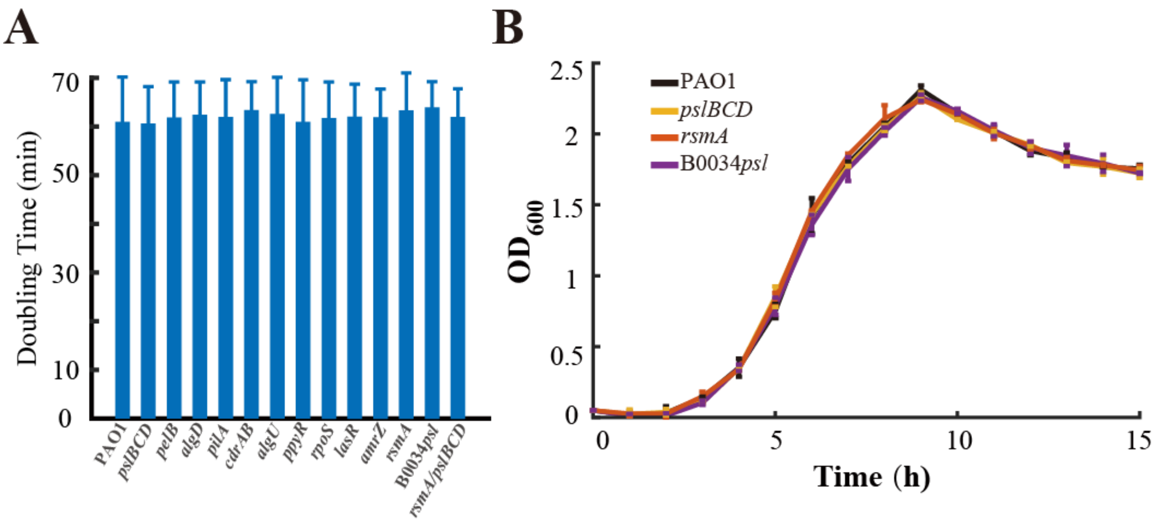

Supplemental Figure 5. Each mutant has no discernable effects on the growth of bacterial cells. **A.** Little changes were observed in doubling time between wild type and each mutant. Data were analyzed from the growth curves of each mutant. **B.** The growth curve was measured in FAB medium with 30 mM glutamate at 37 ℃ for 15 hours. As the shape of growth curves is similar, only four representative curves are depicted for better layout. Error bars represent means ± s. d, and the mean values from two

replicate experiments are represented.

Figure S6

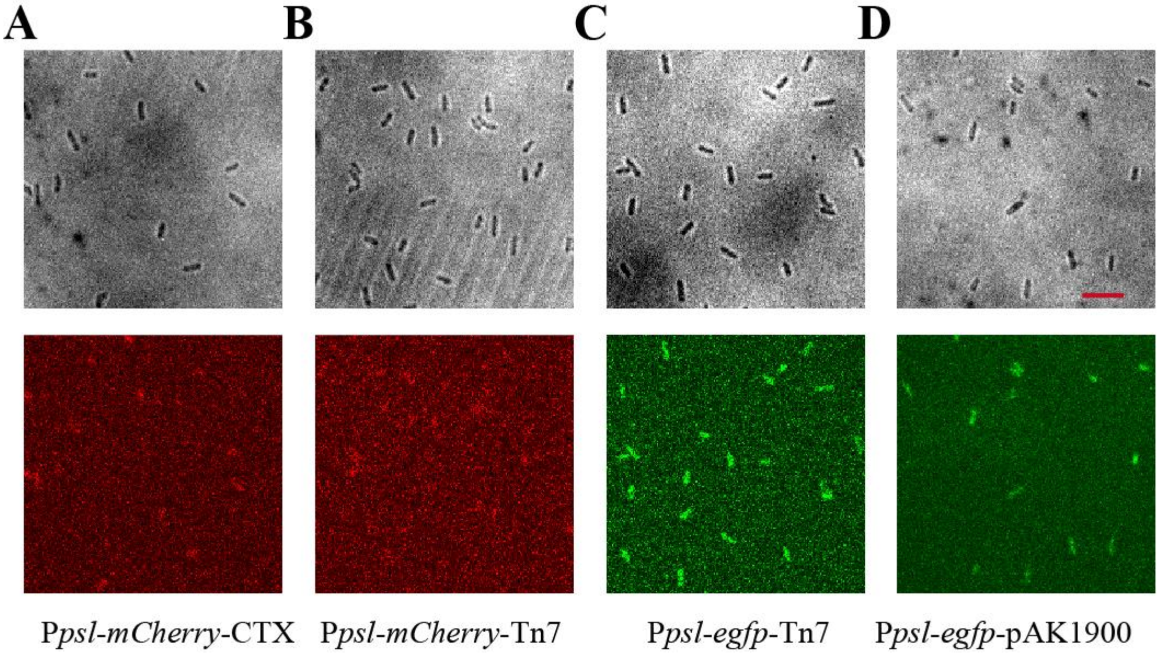

Supplemental Figure 6. Signal of direct expression reporters of *psl* operon is weak. Representative images (31.6 × 31.6 microns) of PAO1 carrying different direct reporters, **A** for P*psl-mCherry*-CTX; **B** for P*psl-mCherry*-Tn7; **C** for P*psl-egfp*-CTX; **D** for P*psl-egfp*-pAK1900. The upper for bright-field images and lower for corresponding fluorescence microscopy images. The signal of chromosomal direct reporter system (CTX and Tn7) are not detectable. In plasmid direct reporter, the fluorescence increased but only a small part of cells could be recognized by fluorescence. The fluorescence micrographs still have a low signal-to-noise ratio and cannot be further analyzed by algorithms. All images are set in a unified scale with a 5 μm bar.

Figure S7

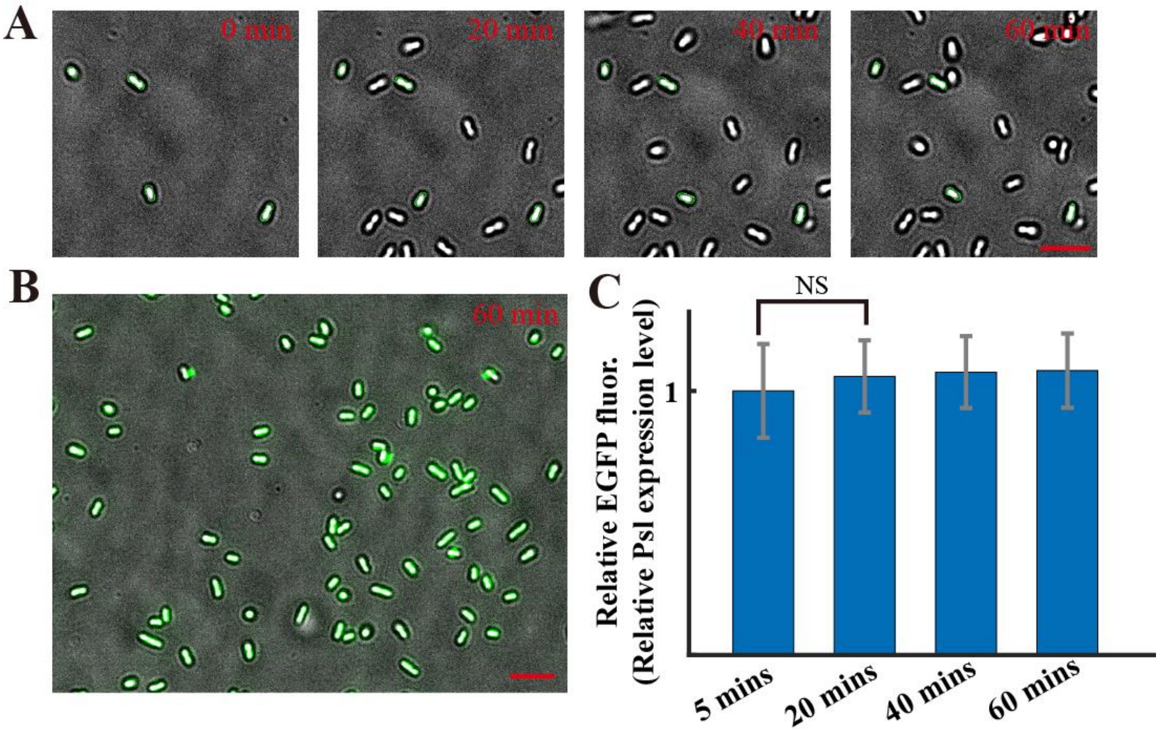

Supplemental Figure 7. Attached cells were tracked to analyze the time duration on surface and the change of fluorescence of the inverted reporter. **A.** Local region of bright-filed images in attaching experiment at different time are depicted. Once cells attached on surface, they rarely detach. The attached cells at t=0 min are masked by green line. **B.** An overlay of bright-field and fluorescence image in attaching experiment at t=60 min. The fluorescence comes from the cells harboring the inverted reporter. **C.** The fluorescence intensity of the attached cells barely changed within 60 minutes. We tracked the attached cells during attaching experiment by bright-field images and the fluorescence micrographs were obtained every 20 minutes to avoid photo-toxicity. We then investigate how the EGFP fluorescence changed for the cells that have attached on the surface in the first 5 minutes. The results show that the fluorescence intensity of the attached cells barely changed within 60 minutes,

which indicates that the attached cells did not have an increase in the expression of Psl during the attaching experiment. In **A** and **B**. scale bar, 5 µm. In **C,** error bars represent means ± s.d (n=3), NS = not significant, (Student's *t*-test).

Figure S8

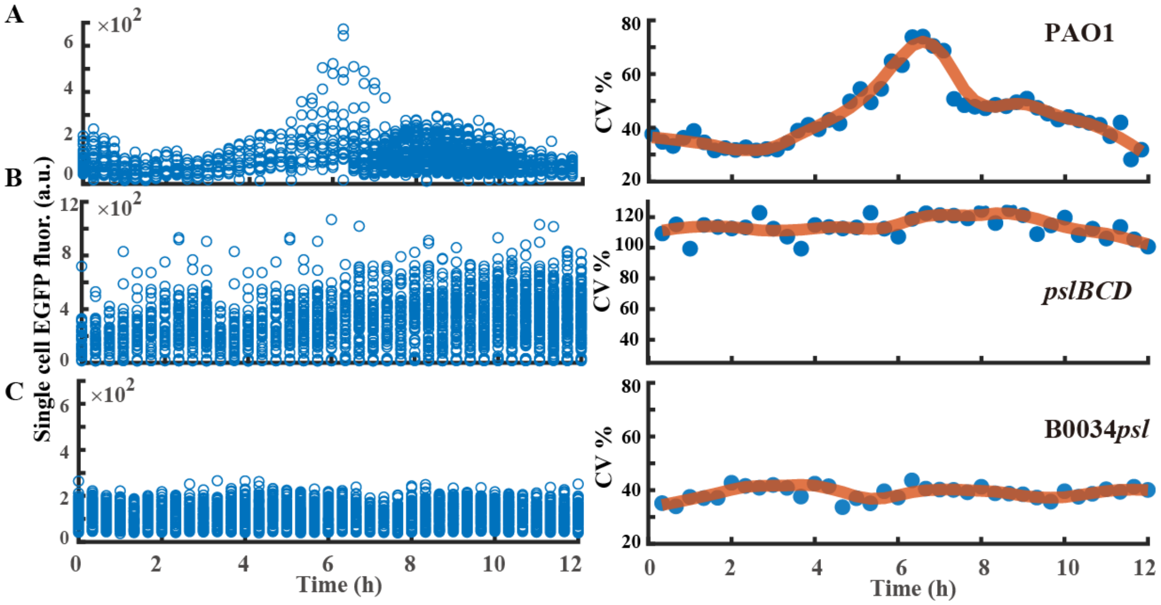

Supplemental Figure 8. Changes of Psl expression in the process of biofilm formation in a flow cell system for PAO1 **(A)**, Δ*pslBCD* **(B)** and B0034*psl*-PAO1**(C)**. PAO1 and Δ*pslBCD* harboring the inverted Psl expression reporter (RYS-2), B0034*psl*-PAO1 harboring RYS-3. The changes are monitored by measuring EGFP fluorescence of surface-attached cells at different time (left panel). The heterogeneity of Psl expression in the population averages were quantified by computing the CV of EGFP fluorescence accordingly (right panel). In wild type, the initially attached cells with higher Psl expression (EGFP intensity lower than 300 (a.u.)) can divide and differentiate into lower Psl expression cells (EGFP intensity greater than 500 (a.u.)), indicated by the CV increased markedly from 37% (0 h) to 78% (6 h). In strain Δ*pslBCD*, the EGFP fluorescence only can report the expression of *psl* operon, because the cells are deficient in production of Psl. Hence, the CV of surface-attached cells keeps large

(110%) over time indicating the heterogeneity expression of *psl* operon. In contrast, the strain B0034*psl*-PAO1, which expressed Psl uniformly, are always have a small CV (around 35%) throughout the experiments, implying small cell-to-cell variability in Psl expression. The range of Y-axis were set identical for better comparison. Experiments were carried out in triplicate and one representative data set is shown.

Figure S9

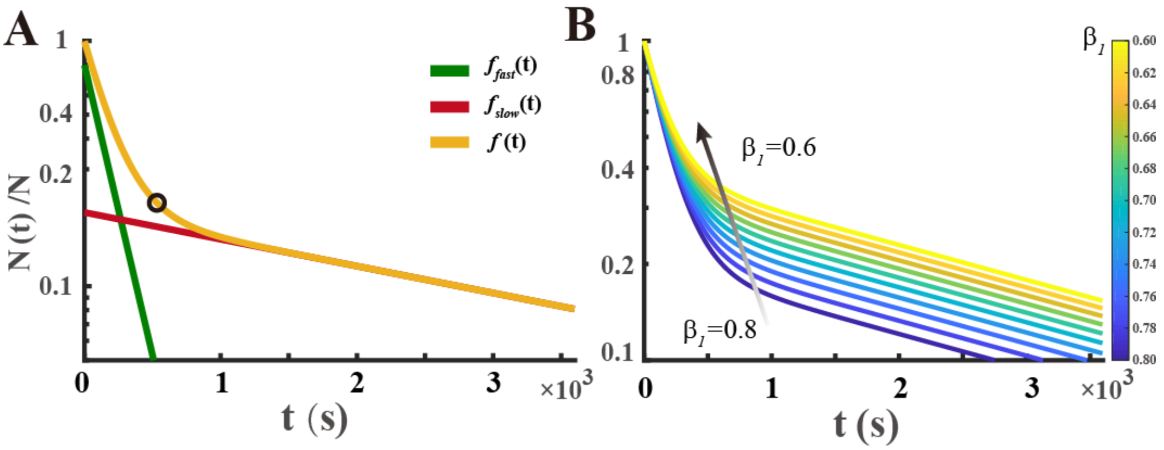

Supplemental Figure 9. A mathematical model was used to illustrate the physics image near the switch point. **A.** Based on the experimental results, an attaching kinetics of planktonic cells were separated into two distinctive types to make the switch point clear (marked by black circle). **B.** To further investigate how the relative proportions of two phenotypes affects the time point of switch, a simulation model was constructed by MATLAB. More information were included in Data Analysis of Supplemental Methods.

Supplemental Methods

Data Analysis

Real time bacterial tracking algorithm using MATLAB (MathWorks) codes(2) allow us to recognize

cell attaching or detaching event in attaching experiments, correspondingly identify division and post-division cell fates (3) and reconstruct the genealogical trees from one mother cell in flow cell experiments. Basically, grayscale images were first converted to binary images for the detection of single cells using image-processing algorithm; The x–y coordinates of the centroids of cell were determined in each frame and subsequently linked into trajectories using particle-tracking algorithm; Single bacterial trajectories were further analyzed using two-point tracking algorithm. More detailed descriptions are following:

Data analysis for attaching experiment

For further analysis to attaching experiments, the variation of number of surface attached cells versus time were generally fitted by two-exponential model using MATLAB from the below equation in which $i = 2$, $N_b^1(0) = N_1$, $N_b^2(0) = N_2$:

$$N_s(t) = N - \sum_i N_b^i(0)\exp(-\alpha_i t)$$

all parameters quoted means the same as indicated in the main text. After capturing the values of parameters from fitting results, the attaching kinetics of planktonic cells ($N_b(t)/N$) were thus described by the following equation in below:

$$N_b(t)/N = N_1/N\exp(-\alpha_1 t) + N_2/N\exp(-\alpha_2 t)$$

but for specified *P. aeruginosa* mutants, such as Δ*pslBCD,* Δ*rsmA,* or B0034*psl*-PAO1, single-exponential model just fits very well and we deemed that only one attaching phenotype exists. Accordingly, the above equation should be modified as follows：

$$N_b(t)/N = N_1/N\exp(-\alpha_1 t)$$

or

$$N_b(t)/N = N_2/N \exp(-\alpha_2 t)$$

The former is appropriate for Δ*rsmA,* or B0034*psl*-PAO1, which are only fast-attaching cells and the later for only slow-attaching strain, such as Δ*pslBCD*. It is noted that attaching kinetics of planktonic cells of different *P. aeruginosa* strains were represented on identical semiology coordinates for easily comparison with each other.

In Figure 1A and Figure 2A, there is a clearly visible switch point that the cells switch from fast to slow attachment. We used a mathematical model to illustrate the physics image near the switch point in the following text.

The Figure 1A and Figure 2A show the attaching kinetics of planktonic *P. aeruginosa* cells ($N_b(t)/N$), and the curves were modeled using an equation in terms of time:

$$N_b(t)/N = N_1/N \exp(-\alpha_1 t) + N_2/N \exp(-\alpha_2 t)$$

Simplifying the above equation yields:

$$f(t) = \beta_1 \exp(-\alpha_1 t) + \beta_2 \exp(-\alpha_2 t)$$

Obviously, the formulation shows that $f(t)$ follows two-exponential decay, in which the shape of curve are determined by 4 terms (α_1, α_2, β_1 and β_2). In our experiments, α_1 and β_1 determine the kinetic of fast-attaching phenotype, which could be described by the equation:

$$f_{fast}(t) = \beta_1 \exp(-\alpha_1 t)$$

Correspondingly, α_2 and β_2 determine the kinetic of slow-attaching phenotype as follows:

$$f_{slow}(t) = \beta_2 \exp(-\alpha_2 t)$$

To make the physics image clear near the switch point, we plotted the curves of $f(t)$, $f_{fast}(t)$ and $f_{slow}(t)$ on the same coordinates where the switch point was marked by black circle. We given $\alpha_1 = 5.45 \times 10^{-3}\ \mathrm{s}^{-1}$, $\alpha_2 = 2.52 \times 10^{-4}\ \mathrm{s}^{-1}$, $\beta_1 = 0.8$ and $\beta_2 = 0.2$, which are the parameters of

wild type in the later exponential phase. Fig. S9A shows that, at the switch point, the fraction of fast-attaching phenotype in bulk has reduced to 5% from 80% (green line), whereas that of slow-attaching phenotype in bulk has only decreased to 18% from 20% (red line). Particularly, in the period before switch point, the steep decrease of cells in bulk comes mainly from the fast-attaching phenotype, because they quickly attached to surfaces; as the number of fast-attaching phenotype decreased over time, the proportion of the slow-attaching phenotype in bulk has overtaken the fast-attaching phenotype and the curve starts a slow-decay near the switch point.

To determine the time point of switch, we simulated a series of curves under different values of β_1 and β_2 by using MATLAB, in which β_1 varied from 0.8 to 0.6, and β_2 varied from 0.2 to 0.4 correspondingly. Fig. S9B shows that switch appears earlier when β_1 became smaller, which is consistent with our experimental results in Figure 1 and Figure 2. That β_1 gets smaller corresponds to a lower fraction of fast-attaching phenotype, which results in that the fraction of slow-attaching phenotype will be over the fast-attaching phenotype in a shorter time.

Therefore, the switch point is mainly determined by the relative proportions of fast- and slow-attaching phenotype in the planktonic cells. Biofilm formation starts with bacterial attachment to surfaces, thus the switch point can affect the number of surface-attached cells in a period of time. Briefly, if planktonic cells have a later switch point, more cells will attach to the surfaces after a certain time, which may subsequently result in micro-colonies formation earlier in the development of biofilms.

Data analysis for flow cell experiment

In flow cell experiments, the cases that one mother cell in frame *i* became two daughter cells in frame *i*+1 were identified as the division events. We assigned each daughter cell to be a new node as a root

identification number for next division, which can be applied for constructing a genealogical tree. Typically, hundreds of division events were caught in one experiment. Post-division cell fates were determined by the analysis of cell behavior in the specified observation window and classified into two types: A post-division cell was defined as one that *stays* if the cell's next division event is still detectable in the field of view. By contrast, a post-division cell was defined as one that *leaves* the vicinity of the division event if its next division was not detected in the field of view. This is similar to the definitions reported previously (3). Note that the cells whose fates are mistakenly regarded as *leaves* type are excluded for the cases when one cell still exists but does not divide before the time point of end frame. Actually, those cells' fates are uncertain.

Data analysis for fluorescent images

For data analysis associated with quantification of fluorescence, we used spinning-disk confocal images to measure the fluorescence in individual bacteria. To compute single cell fluorescence intensity, we subtracted the average fluorescence per pixel of the background from the average intensity per pixel in the given cell and more than 10^4 cells were analyzed for getting mean fluorescence intensity of bacteria populations if necessary. The confocal micrographs acquired in different z-positions were used to reconstruct the three-dimensional (3D) structures of the mature biofilms using volume reviewer plugin of ImageJ *v.1.51w* software.

Supplemental References

1. Kolter R, Siegele DA, Tormo A. 1993. The stationary phase of the bacterial life cycle. Annu Rev Microbiol 47:855-74.

2. Jin F, Conrad JC, Gibiansky ML, Wong GCL. 2011. Bacteria use type-IV pili to slingshot on surfaces. Proc Natl Acad Sci USA 108:12617-12622.

3. Zhao K, Tseng BS, Beckerman B, Jin F, Gibiansky ML, Harrison JJ, Luijten E, Parsek MR, Wong GCL. 2013. Psl trails guide exploration and microcolony formation in *Pseudomonas aeruginosa* biofilms. Nature 497:388-391.
